# Model-based navigation of transcranial focused ultrasound neuromodulation in humans: Application to targeting the amygdala and thalamus

**DOI:** 10.1016/j.brs.2024.07.019

**Published:** 2024-07-31

**Authors:** Mohammad Daneshzand, Bastien Guerin, Parker Kotlarz, Tina Chou, Darin D. Dougherty, Brian L. Edlow, Aapo Nummenmaa

**Affiliations:** a Athinoula A. Martinos Center for Biomedical Imaging, Department of Radiology, Massachusetts General Hospital, Charlestown, MA, USA; b Harvard Medical School, Boston, MA, USA; c Department of Psychiatry, Massachusetts General Hospital, Charlestown, MA, USA; d Center for Neurotechnology and Neurorecovery, Department of Neurology, Massachusetts General Hospital, Boston, MA, USA

**Keywords:** Transcranial focused ultrasound (tFUS), Low intensity focused ultrasound pulsation (LIFUP), Neuronavigation, Acoustic modeling, Hybrid angular spectrum, Neuromodulation

## Abstract

**Background:**

Transcranial focused ultrasound (tFUS) neuromodulation has shown promise in animals but is challenging to translate to humans because of the thicker skull that heavily scatters ultrasound waves.

**Objective:**

We develop and disseminate a model-based navigation (MBN) tool for acoustic dose delivery in the presence of skull aberrations that is easy to use by non-specialists.

**Methods:**

We pre-compute acoustic beams for thousands of virtual transducer locations on the scalp of the subject under study. We use the hybrid angular spectrum solver mSOUND, which runs in ~4 s per solve per CPU yielding pre-computation times under 1 h for scalp meshes with up to 4000 faces and a parallelization factor of 5. We combine this pre-computed set of beam solutions with optical tracking, thus allowing real-time display of the tFUS beam as the operator freely navigates the transducer around the subject’ scalp. We assess the impact of MBN versus line-of-sight targeting (LOST) positioning in simulations of 13 subjects.

**Results:**

Our navigation tool has a display refresh rate of ~10 Hz. In our simulations, MBN increased the acoustic dose in the thalamus and amygdala by 8–67 % compared to LOST and avoided complete target misses that affected 10–20 % of LOST cases. MBN also yielded a lower variability of the deposited dose across subjects than LOST.

**Conclusions:**

MBN may yield greater and more consistent (less variable) ultrasound dose deposition than transducer placement with line-of-sight targeting, and thus could become a helpful tool to improve the efficacy of tFUS neuromodulation.

## 1. Introduction

Transcranial focused ultrasound (tFUS) is an emerging non-invasive neuromodulation approach that allows engagement of deep brain targets with millimeter accuracy [1–5]. The ability of tFUS to reach deep structures makes it highly complementary to transcranial magnetic stimulation (TMS), which can only achieve direct stimulation in the cortex [6]. This is desirable because subcortical nodes of the motor [7], limbic [8,9], and arousal networks [7,10,11] are implicated in the pathogenesis of movement disorders [12,13], psychiatric disorders [14,15], and disorders of consciousness [16,17], respectively. TFUS has been shown to be efficacious in animals [18–26], but human translation has been slow because of the thicker human skull that increases acoustic absorption and scatters acoustic beams. These skull-related effects decrease the acoustic dose in the target region and make it challenging to deliver it to the correct location [27–31]. Although skull effects are recognized as an important consideration in human tFUS [27,29,30,32–34], the majority of human studies using a single focused transducer employ the line-of-sight targeting (LOST) approach for placement. In LOST, the transducer is placed so that the center of the target region, for example the amygdala or the thalamus, lies on the centerline of the device as shown in Fig. S3. LOST placement is challenging and usually requires MRI guidance, which is usually feasible using fiducials markers embedded in the transducer housing. In this approach, the center of the target region is located at a tissue depth corresponding to the focal distance of the transducer in water, which can be achieved using impedance matching pads with appropriate thickness [35,36]. A downside of LOST is that it assumes ideal water propagation and ignores beam scattering and absorption by the skull.

There are several simulation approaches capable of modeling the effects of the skull in tFUS, including spectral and pseudospectral approaches [37–41], finite difference time domain (FDTD) [33,42–45], hybrid angular spectrum (HAS) [32,46–52], finite element modeling [53] and boundary element modeling [54,55], however most of those tools are not easy to use by non-specialists. Some investigators perform a limited number of simulations per subject, often using k-wave [37,38], however setting and solving the subject-specific model requires hours of work, which is feasible for dose verification (one retrospective simulation per subject) but not dose delivery planning (which requires many simulations). K-plan [56] and Sim4Life [57] are commercial software with graphical user interfaces that make it easier to solve the acoustic propagation problem in a subject-specific manner, but those use k-wave and FDTD, respectively, and therefore also suffer from long simulation times that limit their usefulness for dose delivery planning.

In addition to those tools, some investigators have proposed fast simulation strategies for improved dose delivery planning in human tFUS. Yoon et al. [42] proposed a fast FDTD approach whereby the domain is discretized using an adaptive mesh and solved in parallel using a graphical processing unit (GPU), yielding computation times of ~30 s per solve. Shin et al. [58] proposed a super-resolution convolutional neural network combined with a fast low-resolution FDTD solve. Although the investigators report computation times of ~4 s in single-transducer benchmarks, it is not clear how fast this approach can be in practice. Indeed, there is a limit to how low-resolution the initial FDTD solve can be as this technique requires ~10 sample points per wavelength for stability, which represents a minimal resolution of 0.75 mm at 200 kHz and 0.3 mm at 500 kHz. Choi et al. [59] also proposed a neural network for rapid ultrasound beam prediction, but they evaluated the method at 250 kHz where beam aberrations and attenuation by the skull are moderate and easy to model. Importantly, the deep learning models of Shin et al. and Choi et al. are not general and need to be re-trained for new subjects, which takes days. Park et al. proposed a 3D-cGAN neural network for real-time tFUS neuronavigation and showed encouraging results in term of speed and accuracy [60]. However, they used a limited number training inputs (3720), which raises questions about the generalizability of this method to all subjects, transducers and targets. Another method consists in computing the average reflection coefficient of ultrasound waves entering the skull using a 3-layer model of the head and Snell laws [61]. This method provides a more accurate estimate of the ultrasound beam path than LOST, but is based on approximations and was only validated for shallow cortical targets that are easier to reach than the deep sub-cortical nuclei that are the focus of the present study. Another approximate method by the same group consists in performing a time-reversed acoustic simulation from the target region and then placing the transducer at the location corresponding the maximum of a ‘score function’ [62]. A limitation of the methods mentioned in this paragraph is that they are not distributed in an open source manner, which makes them difficult to compare and evaluate objectively.

In this work, we propose a model-based navigation (MBN) approach whereby we pre-compute tFUS beam solutions corresponding to thousands of virtual transducers placed around the scalp of the subject under study. We embed this precomputation approach in a neuronavigation GUI connected to an optical tracking camera, thus allowing real-time visualization of the tFUS beam with a refresh rate of ~10 Hz as the operator freely moves the transducer around the subject’s scalp. Authors have proposed optical tracking approaches for tFUS navigation in the past [63,64], however MBN accounts for bone absorption and scattering in individualized skull models using a validated propagation code. In the current implementation, the pre-computation steps run in ~30 min per subject for a scalp mesh with ~4000 faces and a CPU parallelization factor of 10. If parallelization is not possible, pre-computation time increases to ~1 h for a scalp mesh with ~1000 faces, which still allows acquisition of MRI scans (required for navigation and beam computation) and tFUS sonication in a single study visit. Such rapid pre-computation times are possible thanks to the HAS code mSOUND which solves the generalized Westervelt equation in nonuniform acoustic media in ~4 s per transducer position at 650 kHz on a single CPU (Matlab implementation, 169×169×347 computational grid with resolution $\frac{\lambda }{5}=0.46\;mm$), and has been validated by comparison with other approaches [51,52,65]. We provide open-source, user-friendly graphical user interfaces implemented as Matlab apps for the pre-computation and navigation steps in an effort to promote model-based acoustic dose modeling in tFUS human studies (https://github.com/bastpg).

## 2. Methods

**Fig. 1.**
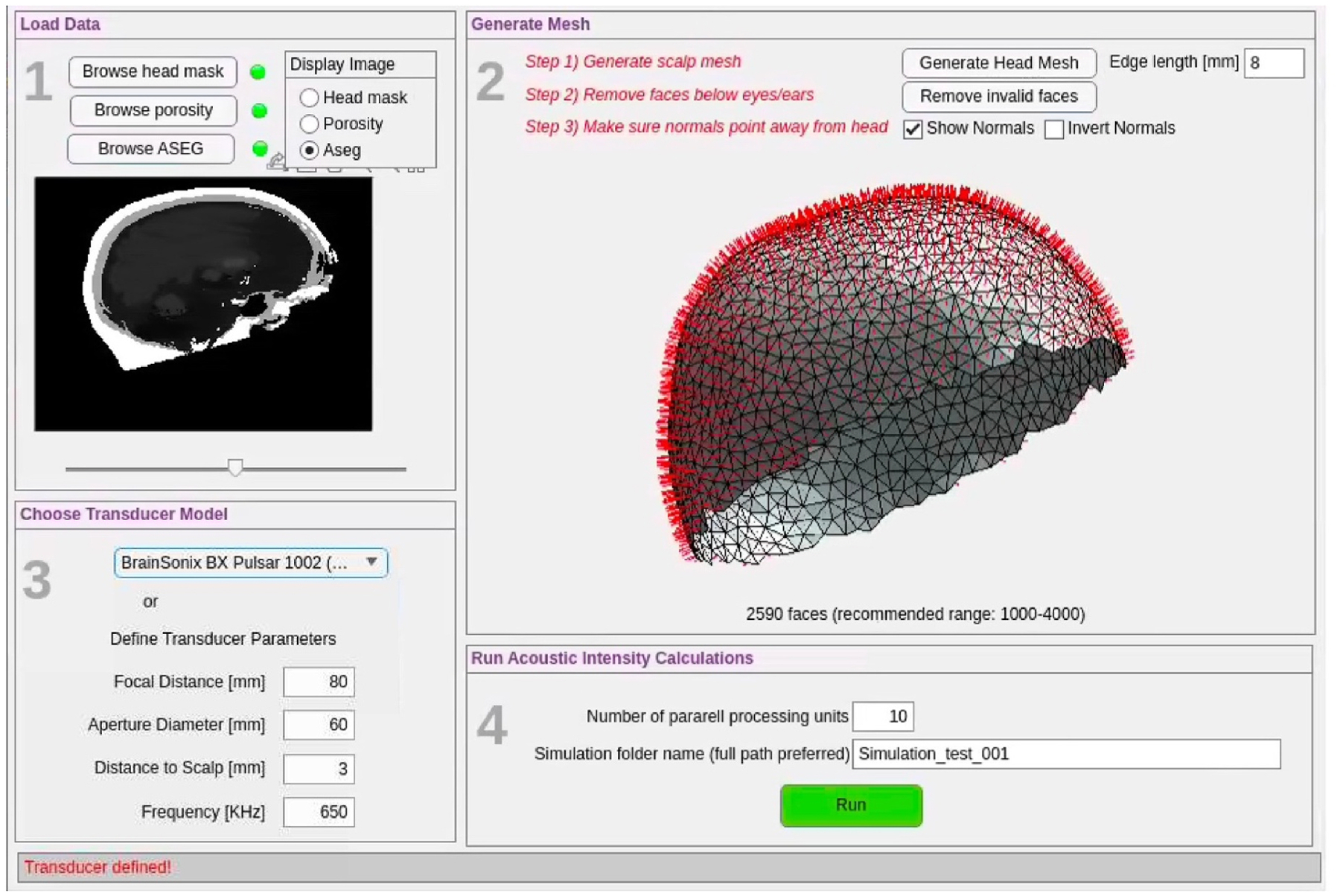
Pre-calculation GUI. **Panel 1:** The operator loads and visualizes the head mask, bone porosity and segmentation index map of deep nuclei structures (‘aseg’ map from FreeSurfer/SAMSEG). Toggling between those maps is useful to check for possible alignment errors. **Panel 2:** A mesh of the scalp is generated and visualized. The operator can adapt the number of faces in the mesh by changing the average triangle edge length and removing faces that do not correspond to valid transducer positions (e.g. below the ears). Faces’ normal are displayed to verify their proper orientation. **Panel 3:** Details of the transducer modeled. **Panel 4:** As the computation proceeds, mSOUND computes beam solutions corresponding to the transducer positions/faces of the scalp mesh in parallel. A typical pre-calculation time is 30 min for 4000 mesh faces with a parallel factor 10.

**Fig. 2.**
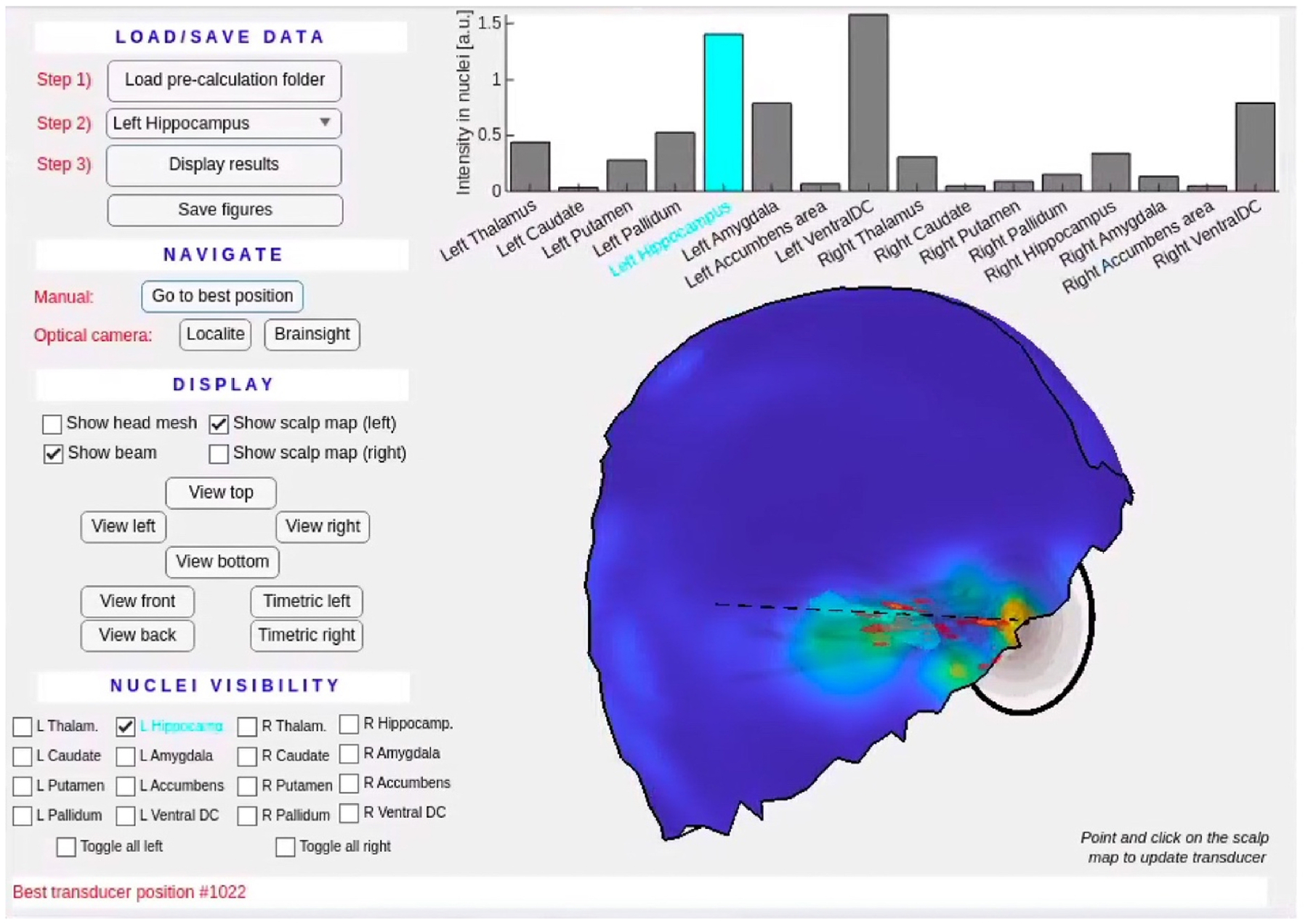
Navigation GUI. The operator loads the results of the pre-calculation, selects the target brain nucleus and can save the various display panels as Matlab figures. The current implementation of the tool uses the so-called ‘aseg’ map generated by Freesurfer and SAMSEG, which supports selection of the L/R accumbens area, amygdala, caudate, hippocampus, pallidum, putamen, thalamus and ventral diencephalon. The ‘Navigate’ panel allows exploring the set of beam solutions by clicking on faces of the scalp map or connecting the tool to a Localite or Brainsight optical tracking camera. There are two main displays on the right-hand side: The top panel is a bar graph of the dose in all the sub-cortical nuclei and the lower panel is the smoothed scalp map with a 3D display of the beam at the current transducer location.

Our model-based navigation (MBN) approach consists of two steps: Pre-computation (Fig. 1) and navigation (Fig. 2). Those steps have dedicated graphical user interfaces (GUI) that are easy to use by non-specialists.

### 2.1. Pre-processing

The first step is to load a head mask, bone porosity map and a segmentation index map of the subject’s head into the pre-computation GUI. The segmentation index map can be computed from a T1-weighted MRI input using Freesurfer [66] or SAMSEG [67] (the so-called ‘aseg’ map), which allows targeting of the L/R accumbens area, amygdala, caudate, hippocampus, pallidum, putamen, thalamus and ventral diencephalon. The porosity map can be estimated by scaling a CT or pseudo-CT volume of the subject, the latter can be computed using a number of methods including atlas-based [68–72], zero echo-time MRI [73–76] or deep learning [77–81]. In this work, we scale Hounsfield units (HU) to acoustic parameters following Aubry et al. [82]:

$$\phi =1-\frac{HU}{1000}$$

$$x=\phi x_{\text{brain}}+\left(1-\phi \right)x_{\text{bone}}$$

where *ϕ* is the bone porosity clipped to [0; 1] and *x* is a generic acoustic variable standing for the density (*ρ*), speed-of-sound (*c*) or acoustic attenuation (*α*). We use bone and brain acoustic properties from the IT’IS material database [83]: $\rho_{\text{brain}},\;\rho_{\text{bone}}=1046,\;1908\frac{Kg}{m^{3}}$, $c_{\text{brain}},\;c_{\text{bone}}=1546,\;3514\frac{m}{s}$ and $\alpha_{\text{brain}},\;\alpha_{\text{bone}}=6.8,\;54.6\frac{Np}{m\times Mhz}$. The head mask is obtained by simple thresholding of the CT (or pseudo-CT) volume. See additional information in supplementary material.

### 2.2. Pre-computation of acoustic beams

In the pre-computation GUI, the head mask is meshed into a model of the subject’s scalp using functions from the iso2mesh toolbox [84,85]. Each face of the scalp mesh represents a virtual transducer location, while the face’ normal represents the transducer normal at that position. The user can refine or coarsen the mesh as needed and exclude faces corresponding to unlikely positions of the transducer, e.g. below the eyes and ears. The total number of faces in the scalp mesh corresponds to the total number of virtual transducer locations solved for, and therefore controls the tradeoff between resolution of the scalp map and computation time. As shown in Figs. S1 and S2, we have found that using 1000 to 4000 virtual transducers (i.e., number of mesh faces) yields reasonable tradeoffs between resolution and computation time.

Next, the operator enters the details of the ultrasound transducer simulated (frequency, aperture diameter, focal distance and distance to scalp, see Fig. 1) into the pre-computation GUI and launches the pre-computation of beam solutions for all virtual transducers in parallel using mSOUND [52,86]. At present, our implementation is limited to virtual transducers located at a fixed distance to the subject’ scalp, which, in practice, corresponds to the thickness of the impedance matching gel pad. Moving the transducer away from the head, i.e. using a thicker impedance matching gel pad, requires solving the entire set of virtual transducer positions anew. We solve beam solutions in a limited computational domain of size 1.3*d* in the transverse direction, where *d* is the transducer aperture diameter, and 2*f* in the longitudinal or z direction, where *f* is the transducer focal distance in water. We found that this was adequate to fully characterize the scattered beam while minimizing computational time (see section 2.5 and Fig. S10). All simulations in this work were performed for the BrainSonix family of transducers (BrainSonix Corp, CA, USA) with focal distances F = 55 mm, 65 mm and 80 mm, 650 kHz operating frequency and 61 mm aperture diameter. See additional information in supplementary material.

### 2.3. Navigation

We developed a visualization GUI shown in Fig. 2 that allows the operator to explore the space of 3D acoustic beam solutions generated in the pre-computation step. The navigation GUI shows the distribution of the dose defined as the sum of the acoustic intensity in the target nucleus for all virtual transducers on the scalp mesh, a metric that we call ‘scalp map’ (one value of the dose per face of the mesh). Scalp maps are helpful to quickly visualize the distribution of the dose across virtual transducer positions. A scalp maps is specific to a subject, transducer and target nucleus; and its peak intensity indicates the transducer position yielding maximum dose deposition for the target nucleus, subject under study. Scalp maps do not provide information about the dose in regions outside the target nucleus however. To address this, we also display the acoustic dose in all nuclei for the selected transducer position, which is helpful to find transducer positions achieving a good balance between maximization of the dose in the target nucleus while minimizating it in other nuclei. For example, a tFUS study attempting to achieve high acoustic dose in the thalamus while minimizing the dose in surrounding nuclei may disfavor lateral beams associated with placement of the transducer on the temporal window, a situation visible in Fig. 9 and S7. In other words, the best transducer position may not always correspond to the maximum of the scalp map, a determination that is best made by the operator. Instead of plotting the raw scalp map, which can have a jagged appearance and yields unstable predictions of the optimal transducer position as a function of scalp mesh resolution, we use a smoothed version that stabilizes the navigation process (Figs. S1 and S2).

**Fig. 3.**
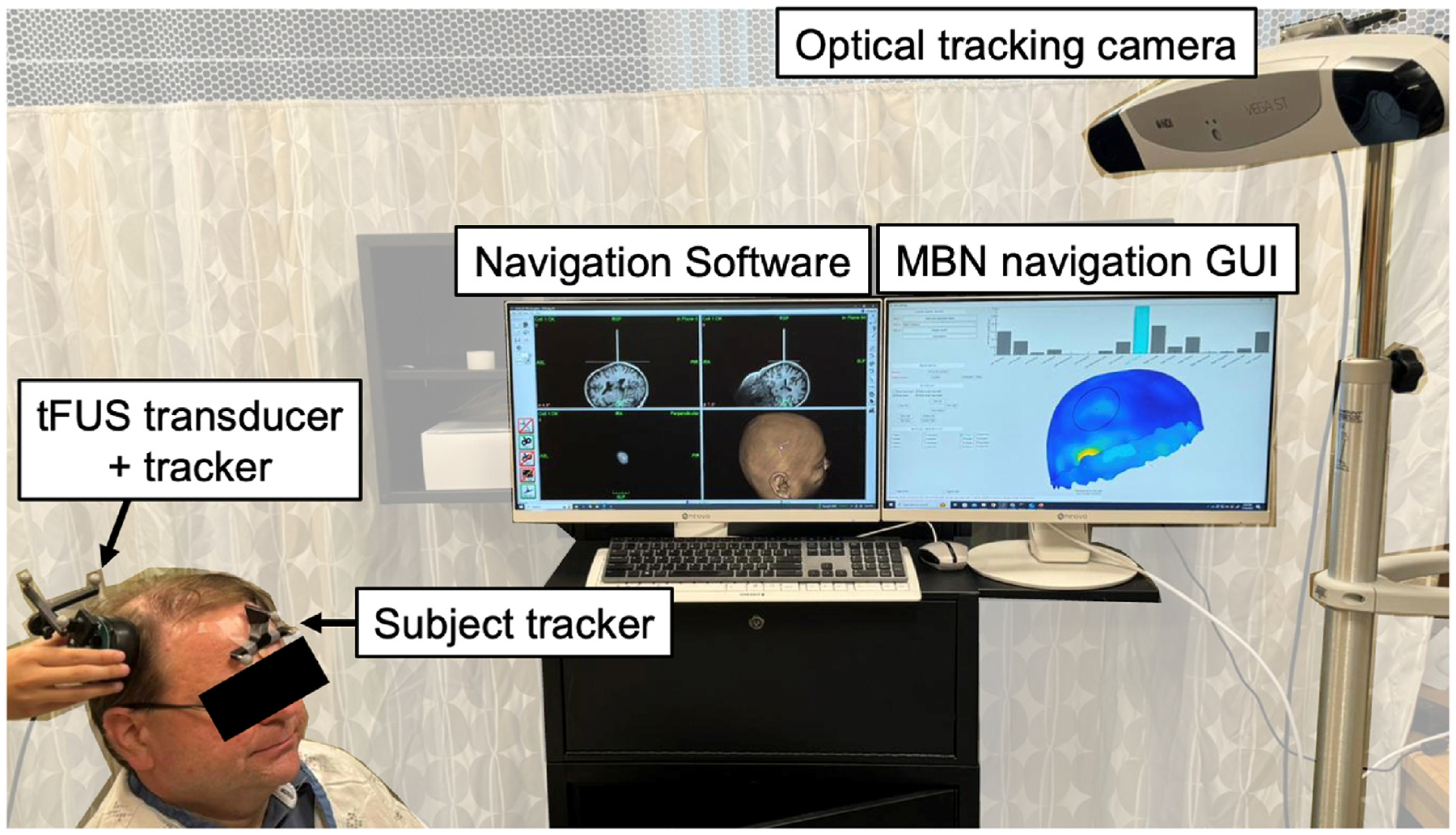
Real-time model-based tFUS navigation with optical tracking. The optical camera tracks both the subject and the transducer and streams this information into the navigation GUI, which allows real-time visualization of the tFUS beam as the operator freely moves the transducer on the subject’ scalp.

Navigation is done either manually, by clicking on a virtual transducer position on the salp map, or automatically by connecting the navigation computer to an optical tracking camera, as shown in Fig. 3. In this work, we use a Polaris Spectra optical tracking camera (Northern Digital Inc, Waterloo, Canada). Both Localite (Localite GmbH, Bonn, Germany) and Brainsight (Rogue Reserach, Cambridge, USA) navigation systems are supported by the navigation GUI. Tracking is performed by mounting a reference tracker on the subject’s head and a coil tracker on the transducer, which allows measuring their relative position and orientation with 2 mm and 1° accuracy. See additional information in supplementary material.

### 2.4. Comparison with line-of-sight targeting (LOST)

We compared dose depositions achieved with MBN and LOST in 13 subjects. We also compared MBN to an approach whereby scalp maps are computed assuming acoustic propagation in uniform water (we call this approach ‘Water’). Additional information in supplementary material.

### 2.5. Comparison of mSOUND with finite difference time domain

See supplementary material.

## 3. Results

**Fig. 4.**
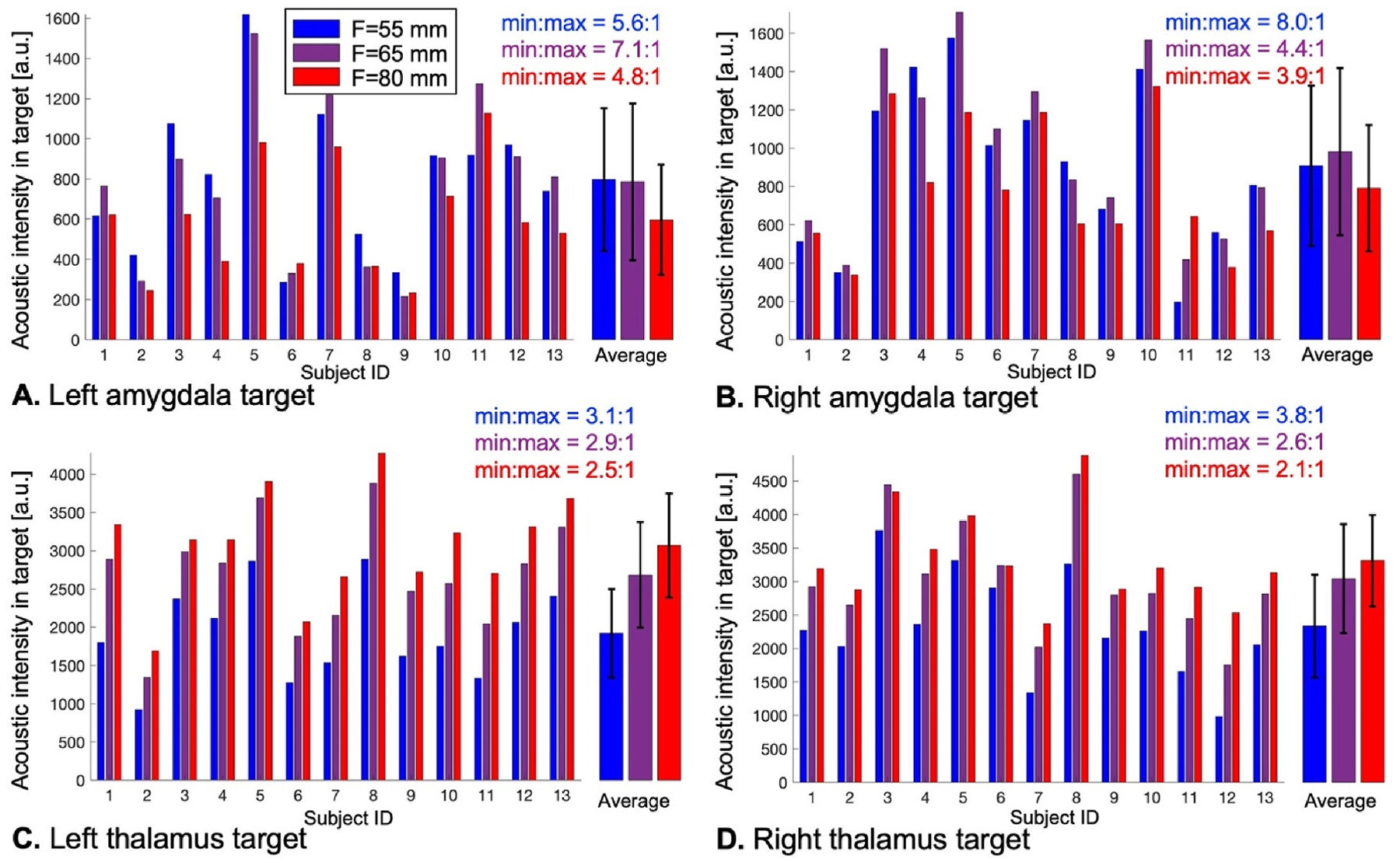
Dose deposited with line-of-sight targeting (LOST) in the left amygdala (A), right amygdala (B), left thalamus (C) and right thalamus (D) in 13 subjects using transducers with focal distances F = 55 mm, 65 mm and 80 mm. The ‘average’ bars show the average and standard deviation of deposited dose values across subjects. The min:max values indicate the range of deposited dose values across subjects for each focal distance (for example, min:max = 10:1 indicates that there is a 10-fold variation of the dose across subjects).

**Fig. 5.**
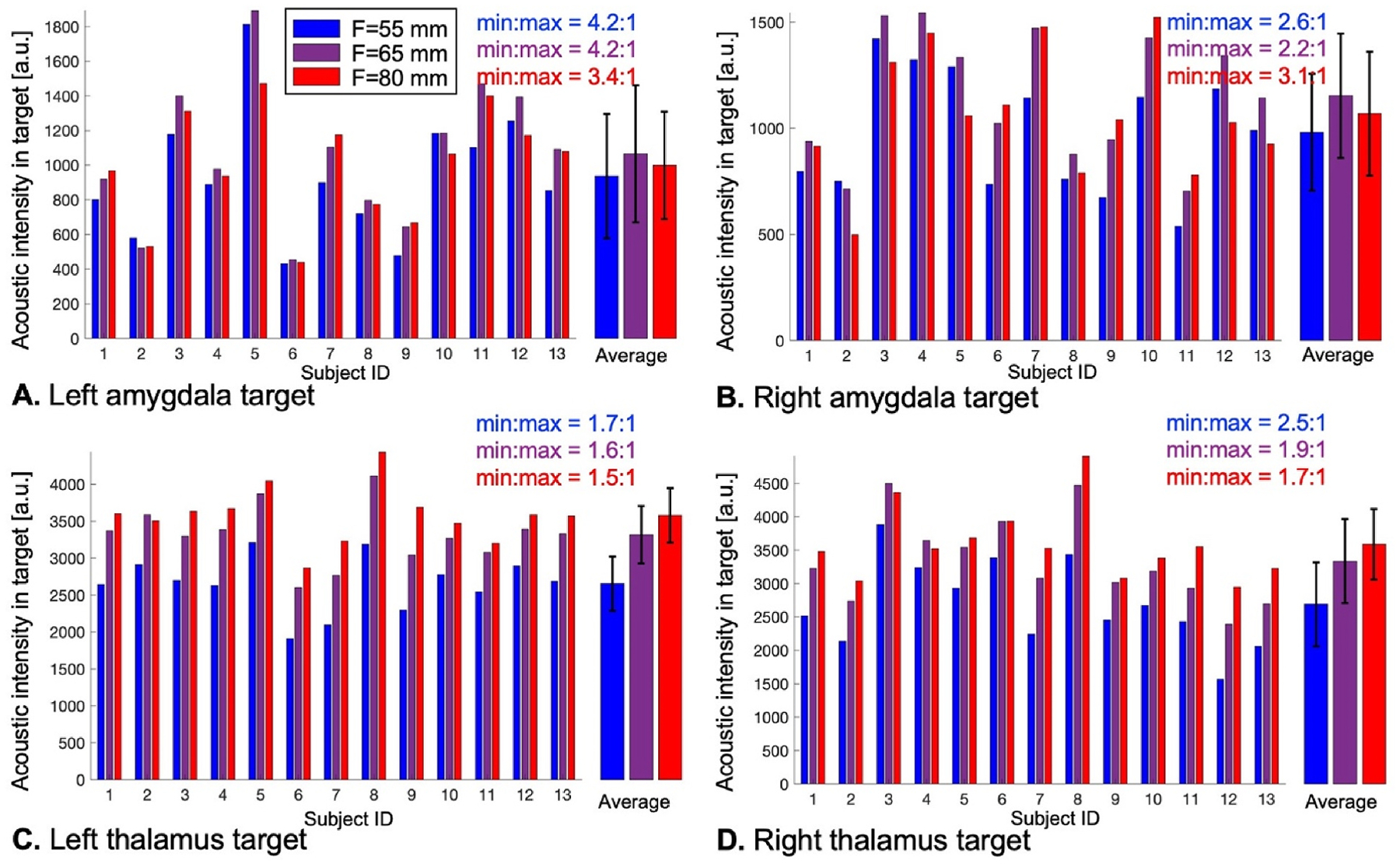
Dose deposited with model-based navigation (MBN) in the left amygdala (A), right amygdala (B), left thalamus (C) and right thalamus (D) in 13 subjects using transducers with focal distances F = 55 mm, 65 mm and 80 mm. The ‘average’ bars show the average and standard deviation of deposited dose values across subjects. The min:max values indicate the range of deposited dose values across subjects for each focal distance (for example, min:max = 10:1 indicates that there is a 10-fold variation of the dose across subjects).

Figs. 4 and 5 show distributions of the acoustic dose in the left/right amygdala and thalamus of 13 subjects using transducer placement optimization with LOST and MBN, respectively, for BrainSonix transducers with focal distances F = 55 mm, 65 mm and 80 mm. Fig. 4 indicates that LOST best reaches the amygdala using the short F = 55 mm focal distance, while the thalamus is best reached using the longer F = 80 mm focal distance. This is not the case with MBN, where the longer F = 80 mm focal distance is optimal (in average for all subjects) both for the amygdala and the thalamus. Another finding is that the distribution of the dose was less variable across subjects with MBN than with LOST: For example, the dose variation was 4.8:1 when targeting the left amygdala with LOST and 2.5:1 when targeting the left thalamus (F=80 mm), whereas it was 3.4:1 and 1.5:1 with MBN, respectively (these represent 34 % and 50 % reductions of the dose variability when using MBN compared to LOST).

**Fig. 6.**
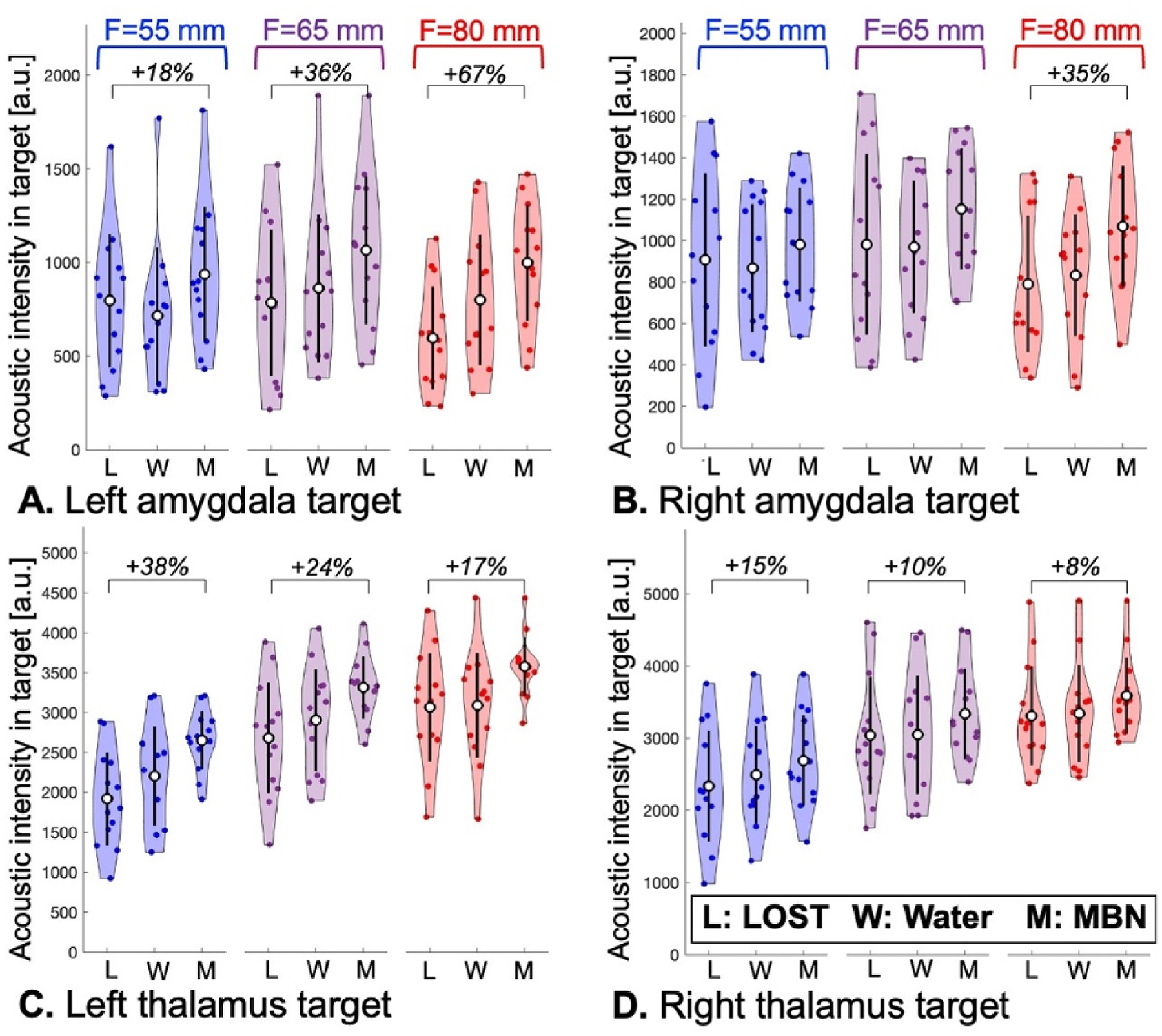
Violin plots showing the distribution, mean and standard deviation of the acoustic dose in the left amygdala (A), right amygdala (B), left thalamus (C) and right thalamus (D) when using line-of-sight targeting (LOST), water simulation (Water) and model-based navigation (MBN) for transducers with focal distances F = 55 mm, F = 65 mm and F = 80 mm. Brackets indicate statistically significant increases of the deposited dose with MBN compared to LOST (two-sided *t*-test, p = 0.05).

The distributions of LOST, ‘Water’ and MBN doses in the left/right amygdala and thalamus of the 13 subjects are compared in Fig. 6. In average, MBN increased the acoustic dose by 8–67 % compared to LOST, yielding statistically significant differences in all simulated scenarios except when targeting the right amygdala with the F = 55 mm and F = 65 mm transducers. The ‘Water’ dose distribution sits roughly between MBN and LOST, which is intuitive since ‘Water’ considers all possible transducer positions and is more general than LOST, but does not model skull effects.

**Fig. 7.**
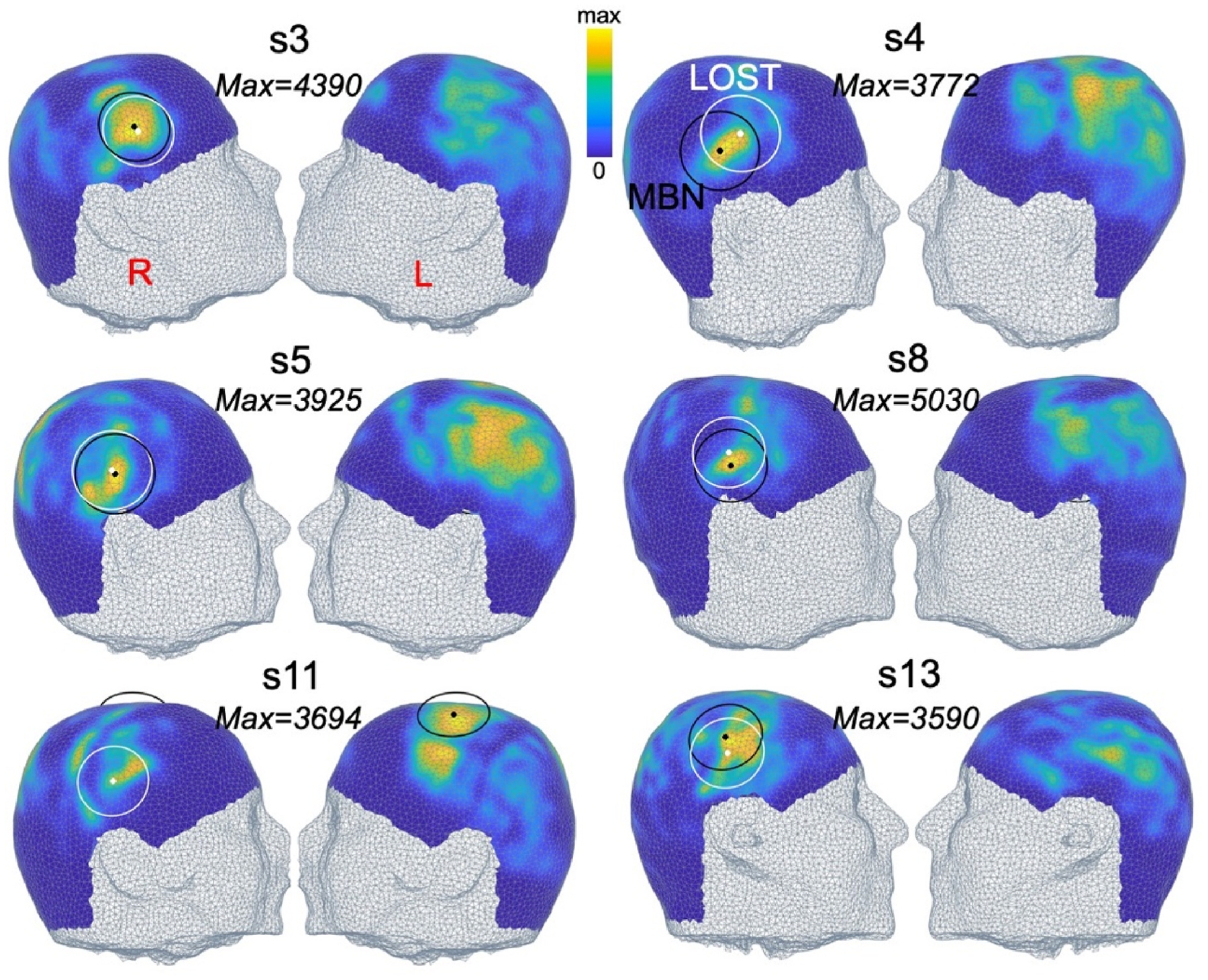
Model-based navigation (MBN) scalp maps targeting the right thalamus with the F = 80 mm transducer. The value next to each map indicates the peak dose deposition in the target at the optimal MBN transducer position. Colormaps are scaled to that value. Optimal MBN transducer locations are shown in black, LOST locations in white.

**Fig. 8.**
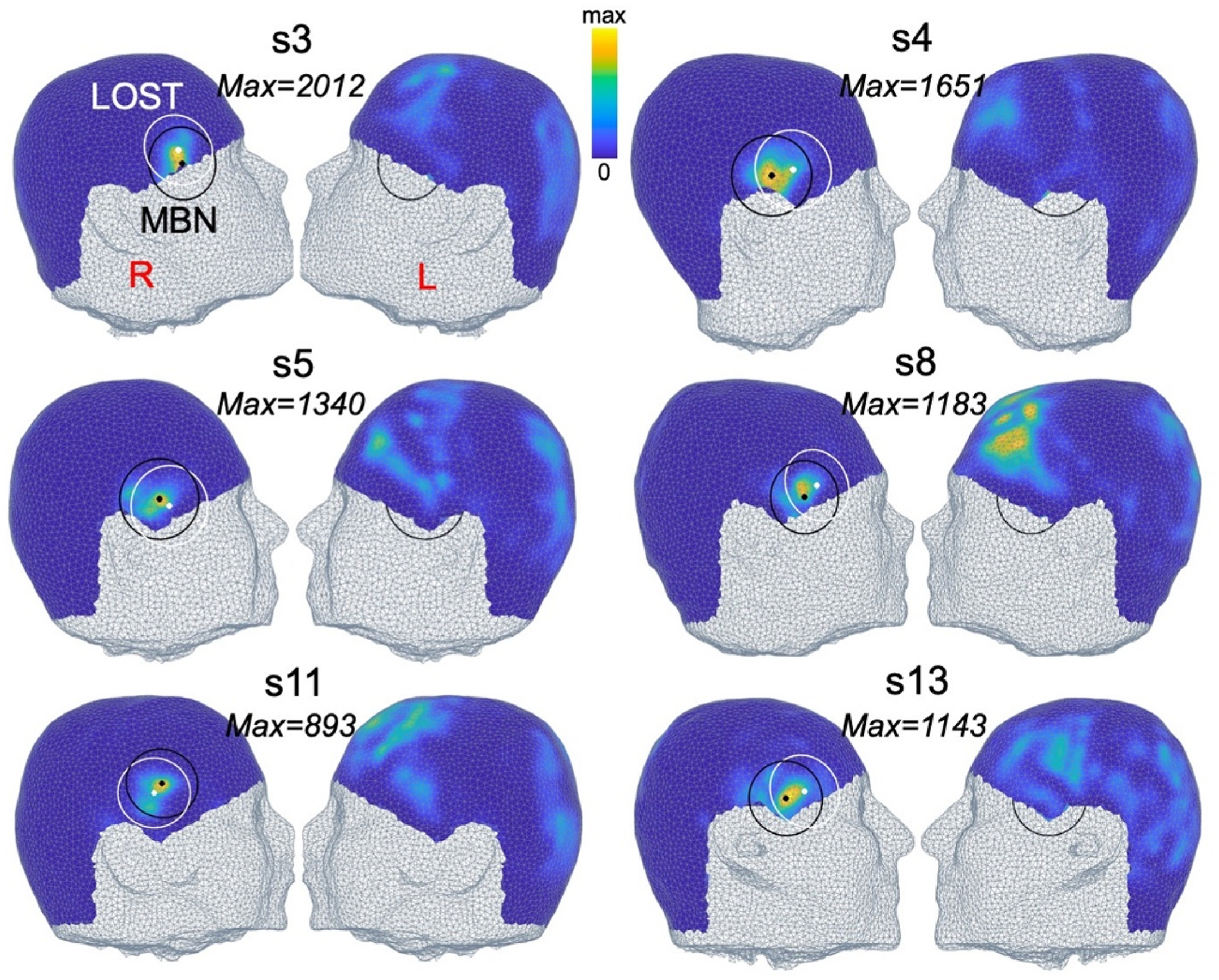
Model-based navigation (MBN) scalp maps targeting the right amygdala with the F = 80 mm transducer. The value next to each map indicates the peak dose deposition in the target at the optimal MBN transducer position. Colormaps are scaled to that value. Optimal MBN transducer locations are shown in black, LOST locations in white.

Figs. 7 and 8 show MBN scalp maps associated with the right thalamus and amygdala and the F = 80 mm transducer in 6 of the 13 subjects (scalp maps for the left thalamus and amygdala are shown in Figs. S5 and S6). Those maps vary significantly between subjects, especially for the thalamus target where the optimal MBN location is sometimes on the temporal window (Fig. 7, subjects 3, 4, 5 and 8), a location used in many LOST studies, and sometimes in more superior locations (Fig. 7, subjects 11 and 13). Optimal MBN locations for the amygdala target were more consistent across subjects because this nucleus is shallower and less central than the thalamus (i.e., closer to the temporal lobe), thus always yielding optimal positions on the ipsilateral temporal window.

**Fig. 9.**
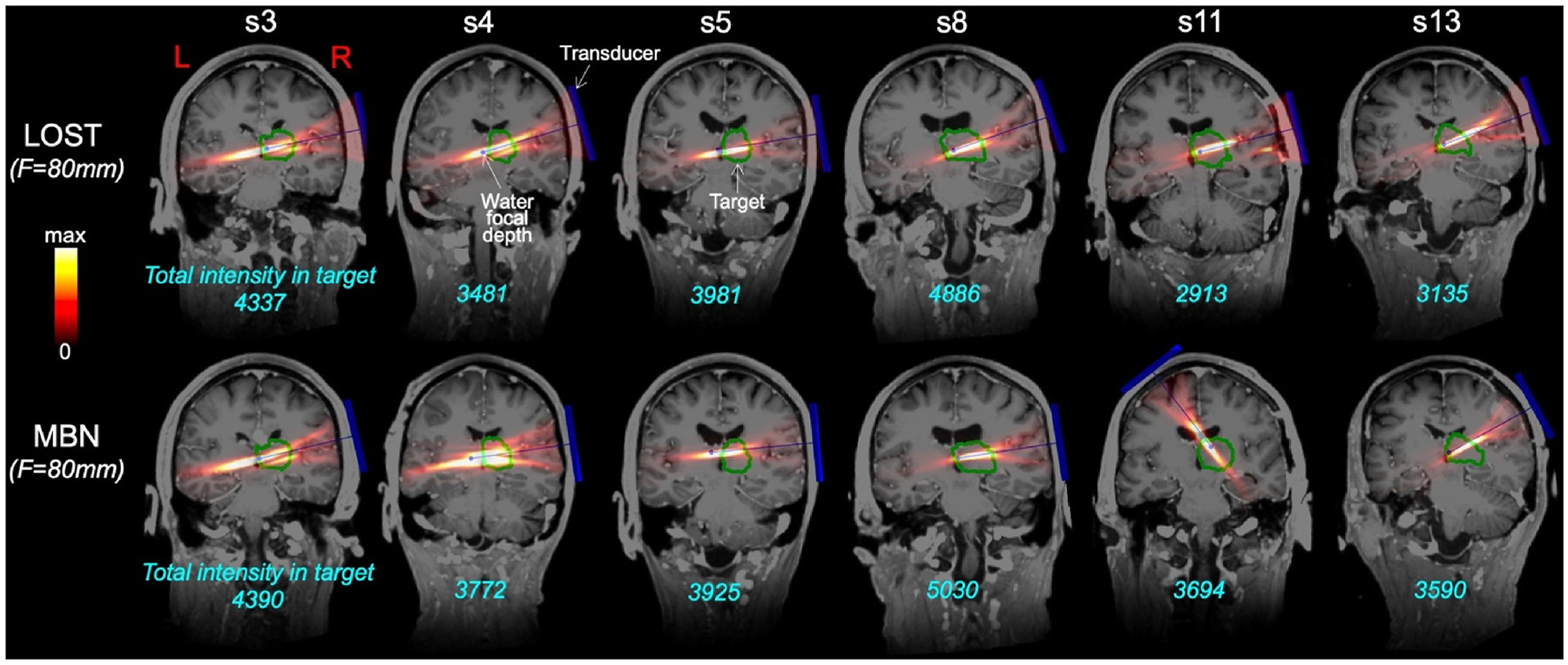
TFUS beams targeting the right thalamus, overlaid on T1-images for 6 of the 13 subjects in this study. The first and second rows show beam solutions associated with line-of-sight targeting (LOST) and model-based navigation (MBN), respectively. The transducer is shown in dark blue (the transducer centerline ends in a small dot indicating the water focal distance, which is F = 80 mm both for LOST and MBN since this is the optimal configuration for this target as shown in Figs. 4 and 5). The outline of the target nuclei is in green. The cyan numbers indicate the dose deposited in the target region in arbitrary units.

**Fig. 10.**
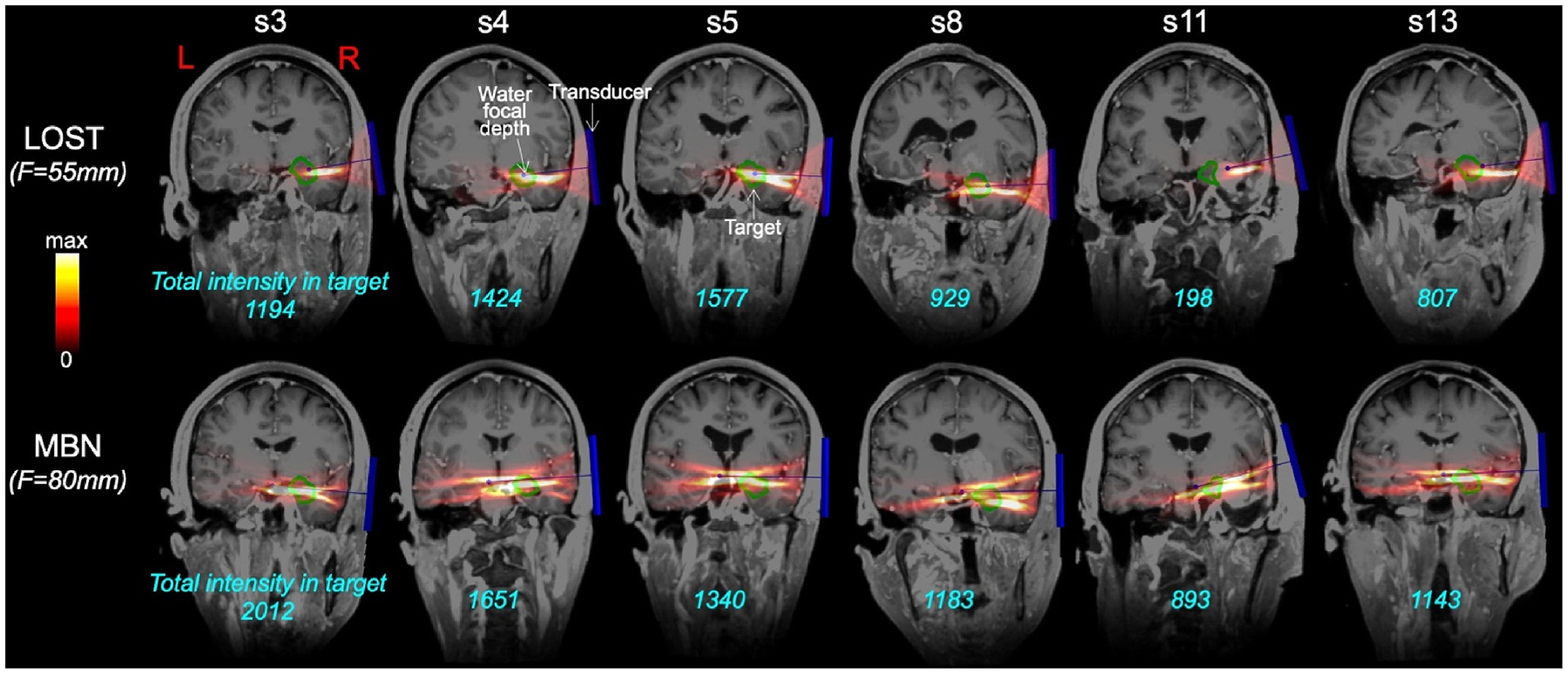
TFUS beams targeting the right amygdala, overlaid on T1-images for 6 of the 13 subjects in this study. The first and second rows show beam solutions associated with line-of-sight targeting (LOST) and model-based navigation (MBN), respectively. The transducer is shown in dark blue (the transducer centerline ends in a small dot indicating the water focal depth, which is F = 55 mm both for LOST and F = 80 mm for MBN since these are the optimal configurations for this target as shown in Figs. 4 and 5). The outline of the target nuclei is in green. The cyan numbers indicate the dose deposited in the target region in arbitrary units.

Figs. 9 and 10 show MBN and LOST beams targeting the right amygdala and right thalamus overlaid on T1 MRI images for 6 of the 13 subjects (left thalamus and left amygdala beams are shown in Figs. S7 and S8). Those indicate that LOST, when properly implemented, generally does a good job at targeting the thalamus and amygdala, with only a few complete misses (subjects 3 and 11 in Fig. 10, subjects 11 and 13 in Fig. S8). MBN avoids those complete misses and further increases the dose in all scenarios.

## 4. Discussion

### 4.1. Model-based navigation for transcranial focused ultrasound

We propose a modeling-based navigation (MBN) approach for optimal placement of transcranial focused ultrasound (tFUS) transducers that accounts for skull-induced beam aberration and scattering. The approach combines pre-calculatation of acoustic beams at thousands of virtual locations with optical tracking of the transducer’s position and orientation in 3D space, thus enabling real-time visualization of the tFUS beam as the operator freely moves the transducer on the subject’s scalp. We provide acoustic dose deposition metrics to aid navigation, including ‘scalp maps’ representing the distribution of the acoustic dose in the target nucleus for all virtual transducer locations. Scalp maps are specific to a target nucleus, subject and transducer and allow quick determination of transducer locations yielding high dose deposition in the region of interest. A major goal of this work is to facilitate the deployment of state-of-the-art tFUS modeling in human studies, which we believe is critical for successful translation of tFUS into humans. For this reason, we provide Matlab GUIs that are easy to use by non-specialists without a computational background which we distribute in an open-source manner (https://github.com/bastpg).

### 4.2. Comparison of MBN with LOST

We simulated tFUS scenarios targeting the amygdala and the thalamus in 13 subjects. Those simulations indicate that MBN improves tFUS targeting by 8–67 % compared to line-of-sight targeting (LOST) and avoids complete target misses that otherwise affect 10–20 % of LOST cases. We also found that variation of the dose across subjects was smaller with MBN than with LOST. Specifically, the LOST dose varied 4.8-, 3.9-, 2.5- and 2.1-fold across subjects when targeting the left amygdala, right amygdala, left thalamus and right thalamus, respectively, with the F = 80 mm transducer. In contrast, the MBN dose variation across subjects was 3.4-, 3.1-, 1.5- and 1.7-fold for those targets. Large variations of the acoustic dose are caused by variations of the skull porosity and geometry across subjects that are accentuated by inconsistent transducer positioning when using LOST. Greater and more uniform dose delivery across subjects using MBN is desirable as this may lead to greater efficacy and reduced variability of clinical outcomes.

**Fig. 11.**
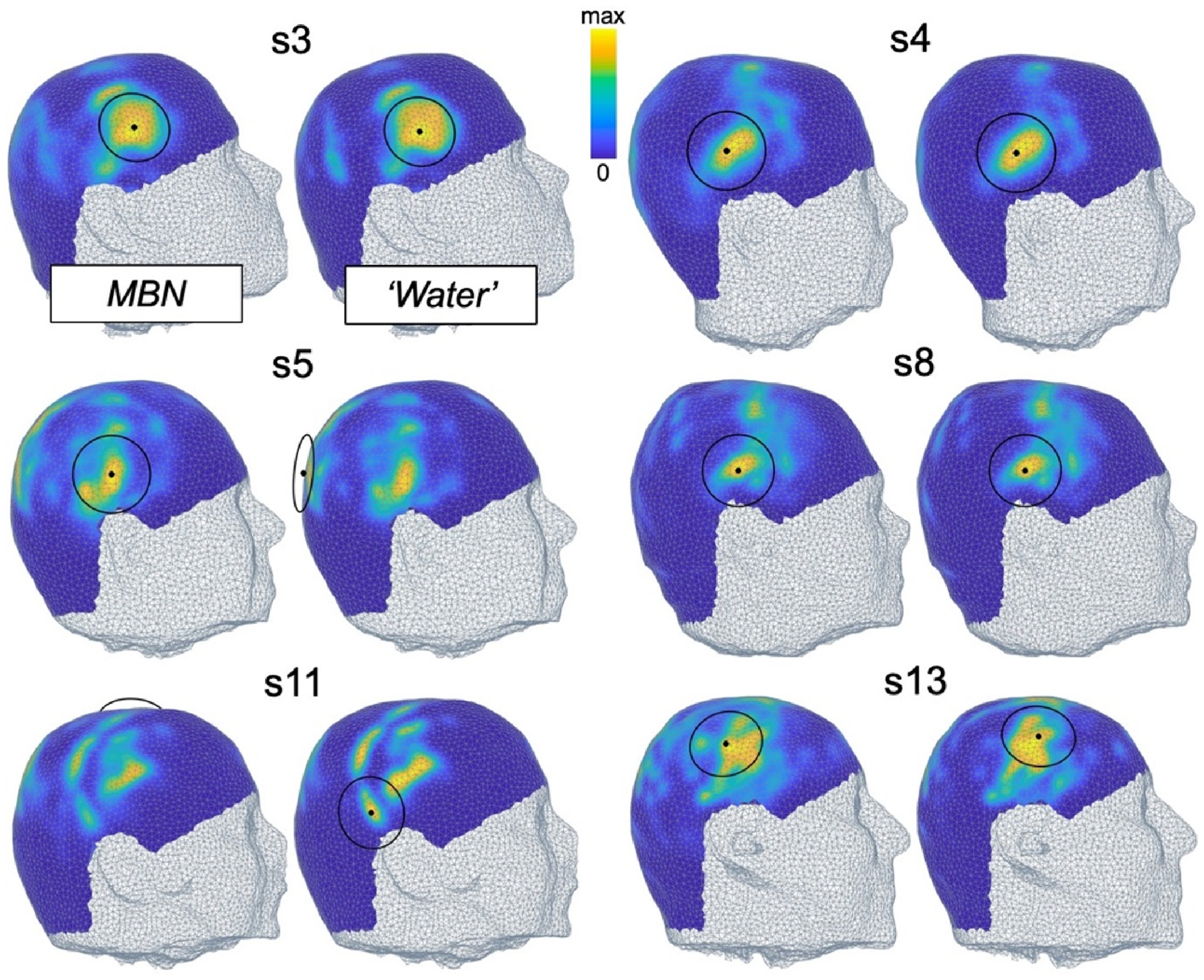
Scalp maps of the dose in the right thalamus for 6 of the 13 test subjects, obtained with modeling of the skull (model-based navigation, MBN) and without modeling of the skull (water simulation, ‘Water’). In the ‘Water’ approach, the ideal beam profile of the transducer in water is overlapped onto the geometry of the head of the subject for all virtual transducer positions. Therefore, ‘Water’ can be viewed as a generalization of LOST. The transducer modeled has a focal distance F = 80 mm. Optimal transducer locations corresponding to the peak of those maps are shown in black.

We found that scalp maps varied greatly across subjects and target nuclei, which shows that tFUS dose delivery planning should be done in a study- and subject-specific manner. We studied two brain targets in this work: The amygdala, which is small and lateral, and the thalamus, which is larger and located centrally in the brain. Thalamus scalp maps displayed broad transducer placement regions associated with high dose deposition, indicating that there is some latitude in the exact placement of the transducer and that targeting is relatively robust to unavoidable navigation errors. In contrast, amygdala scalp maps were sparse with narrow regions associated with high dose deposition, making precise positioning and navigation more critical in this case. See additional discussion on MBN vs. ‘Water’ in supplementary material (Fig. 11).

### 4.3. Quantitative dose calibration

In addition to dose delivery planning, MBN provides dose metrics that could be helpful to explain the variability of clinical and neuroimaging (fMRI, EEG etc.) outcome measures [87–92]. In this work we report the uncalibrated acoustic dose intensity in arbitrary units for the commercial BrainSonix transducers [93]. System-specific calibration to absolute units (W/m^2^) requires mapping the transducer beam in water using a hydrophone, a step that needs to be performed by the operator. Absolute calibration of dose metrics in W/m^2^ is essential for assessment of thermal and mechanical effects but not so much for dose delivery planning (i.e. Where should the transducer be placed?) since such a calibration factor applies to all transducer positions and all subjects in the same way.

Subject-specific computation of the thermal safety index could be achieved by adding a thermal solver to our suite of tools, for example the open-source Pennes bioheat solver in k-wave. Thermal simulations are slow, therefore this step may only be performed for the final, optimal transducer location. The mechanical index on the other hand, i.e. the ratio of peak negative pressure (in MPa) to the square root of the frequency (in MHz), is trivial to compute and could easily be displayed as an additional map provided that the operator supplies the acoustic intensity calibration factor. Subject-specific calculations of thermal and mechanical indices allow assessing safety in individual subjects, which could possibly lead to less constraining, but still safe, acoustic intensity limits in human studies.

### 4.4. Definition of target nuclei

MBN requires an ‘aseg’ segmentation map of sub-cortical nuclei from Freesurfer [66] (~2 h per subject) or SAMSEG [67] (~13 min with a parallelization factor of 10) which supports targeting of the L/R accumbens area, amygdala, caudate, hippocampus, pallidum, putamen, thalamus and ventral diencephalon. In the future, it could be valuable to include sub-segmentation atlases of the thalamus [94], amygdala [95], brainstem [96] and hippocampus [97] in MBN since the resolution of tFUS may permit targeting sub-regions of those nuclei. Another idea could be to add white matter fiber atlases derived from subject-specific [98–100] or population average diffusion MRI data [101] into MBN, which may eventually be of interest as white matter targets have been shown to be effective for the treatment of psychiatric disorders using deep brain stimulation [102–107].

### 4.5. Computational times and requirements

Computing power and memory requirements are moderate for MBN as modern personal computers and laptops often come standard with 4–8 CPU cores and 4 GB of RAM or more, which is enough to run MBN in the most basic configuration (1 h pre-computation, 1000 virtual transducers, 1 CPU). Of course, the more CPU cores, the better (computation time decreases to 30 min for 4000 virtual transducers using 10 CPUs). Such short pre-computation times are feasible thanks to the speed of mSOUND, which runs in ~4 s per virtual transducer, and allow conducting the MRI examination and tFUS sonication portions of a study in a single visit. By comparison, k-wave and GPU-accelerated FDTD run in ~15 min and ~2 min, respectively, which would yield days of pre-computation for the entire set of virtual transducers even when distributing the calculation on multiple cores. To minimize computation time, we run mSOUND in a reduced computational domain of size 1.3*d* in the transverse direction and 2*f* in the longitudinal direction, where *d* and *f* are the aperture diameter and the focal distance of the transducer, respectively. Such computational domain size prohibits modeling of internal reflections and standing wave effects; however Fig. S10 shows that this does not significantly impact the accuracy of hotspot position and shape predictions at 650 kHz. See additional discussion in supplementary material.

### 4.6. Smoothing of scalp maps

See supplementary material.

### 4.7. Validation of MBN predictions

This work was an simulation study that needs to be validated by comparison with experimental data. The mSOUND solver is a validated tool [51,52,65] and the largest source of error in our predictions likely originates from inaccurate estimation of acoustic properties from CT or pseudo-CT data. This is a difficult and active area of research, and our tool reflects the state-of-knowledge in the field. For example, CT scans at clinical resolutions do not allow characterization of the geometry of pores in cortical bone, an information that is needed for accurate modeling of ultrasound scattering through the skull. Using additional metrics such a T2-weighted MRI or ultrasound transmission measurements may help improve modeling accuracy, however such considerations are beyond the scope of this work [29]. As the field progresses, we will update our tool so that it reflects the state-of-the-art. A reassuring fact is that acoustic absorption, which is difficult to estimate accurately, mainly affects absolute dose quantification [108] whereas the speed-of-sound, which is easier to estimate from Hounsfield units, is the main determinant of hotspot position and shape [31]. Therefore, MBN predictions of optimal transducer locations are likely reasonably accurate, but quantitative dose predictions across subjects should be interpreted with caution. See additional discussion in supplementary material.

## Supplementary Material

1
