# Supplementary material for: Model-based navigation of transcranial focused ultrasound neuromodulation in humans: Application to targeting the amygdala and thalamus
Source: Brain Stimul. Author manuscript; Available in PMC 2024 Sep 2. (PMC11367617; doi:10.1016/j.brs.2024.07.019)
Supplement: 1 [file NIHMS2018136-supplement-1.docx]

**Supplementary material: Methods**

- 1. ***Pre-processing***

We recommend that the input T1 map be pre-processed following steps 1-11 of FreeSurfer’s ‘recon-all’ script, which includes transformation into a 1 mm^3^ 256x256x256 image grid, non-uniform intensity correction, computation of the Talairach transform, intensity normalization, skull-stripping and segmentation of white matter and cortical and deep nuclei grey matter structures^66^.

- 1. ***Pre-computation of acoustic beams***

In short, mSOUND is a modified HAS method for fast acoustic propagation in strongly heterogeneous media that has been validated extensively by comparison with other propagation codes^51,52,65^. Like all HAS methods, mSOUND propagates the acoustic pressure field along the z-dimension of the input image stack, which means that for each virtual transducer the input acoustic parameter maps must be interpolated in that transducer’s frame of reference. This interpolation step is implemented in C++ in the pre-computation GUI for maximum speed. We discretize the simulation domain using a uniform Cartesian grid with resolution $\frac{\lambda}{5}$, where $\lambda$ is the wavelength in water. The fact that mSOUND is stable with such coarse discretization contributes to the fast pre-computation time. Reflections at the computational domain boundaries are minimized using a non-reflecting layer with parameters $\alpha=0.3$ and $\gamma=0.1$^87^. We compute the acoustic intensity from the pressure output of mSOUND ($P$) using the plane wave approximation: $I=\frac{\left| P \right|^{2}}{2\rho c}$, where $\rho$ is the density of the medium and $c$ is the speed of sound. To reduce disk space requirements, we do not save the 3D intensity maps for all virtual transducers. Instead, we save the values of the dose deposited in all nuclei (i.e. in this work we define the acoustic dose as the sum of the acoustic intensity in the target nucleus) as well as a compact surface-mesh representation of beam profiles for visualization purpose.

- 1. ***Navigation***

When using the Localite navigation system, the tracking camera and navigation GUI communicate via a TCP/IP protocol managed in Matlab, yielding a display refresh rate of ~10 Hz. The bottleneck in this process is the slowness of the Matlab visualization and faster refresh rates could be achieved using more efficient graphics, for example with 3D Slicer^60,87^. When using Brainsight, streaming of the transducer 3D coordinates and orientation into the navigation GUI is performed by reading a text file updated in real-time by the navigation system.

- 1. ***Comparison with line-of-sight targeting (LOST)***

The 13 test subjects are part of a database that was previously used to create a pseudo-CT reference atlas^68^ and includes MRI T1-weighted MPRAGE (3T MAGNETOM Trio, Siemens Healthineers) and CT scans (LightSpeed QX/I; GE Healthcare) acquired at the Massachusetts General Hospital. LOST was implemented as a Matlab GUI shown in Fig. S3. The LOST GUI allows 3D placement of the transducer on the subject’ scalp as well as visualization of the transducer centerline and scalp normal, thus allowing control of the incident angle of the acoustic beam in the head (≤10° in this work). We also compared MBN to an approach whereby scalp maps are computed assuming acoustic propagation in uniform water. This ‘Water’ approach, like LOST, does not simulate skull effects, but is more general than LOST as it overlaps the ideal beam profile of the transducer onto the geometry of the head for all virtual transducers, whereas LOST only considers a small number of locations.

- 1. ***Comparison of mSOUND with finite difference time domain***

As explained in section 2.2, mSOUND is run in a reduced computational domain in order to limit computational time and requirements. Doing so prevents modeling internal reflections on the walls of the skull that can lead to standing wave patterns. A previous simulation study found that standing waves are more pronounced at low frequencies (250 kHz vs. 500 kHz in Ref. 44) and in small skulls such as those of monkeys and that, overall, this effect has a minimal impact on tFUS hotspots shapes and positions in humans. To confirm those conclusions, we compared mSOUND simulations in subject #1 (F=80 mm transducer, LOST transducer placement targeting the left/right amygdala and left/right thalamus) with a validated finite difference time domain (FDTD) code^42^. FDTD simulations were performed with propagation times associated with one pass of the wave through the skull cavity (‘No reflection’), and two round trips of the wave inside of the skull cavity (‘With reflections’). The ‘No reflection’ simulated was associated with an FDTD propagation duration of T=107 μs, while the ‘With reflections’ was associated with an FDTD propagation duration of T=428 μs. FDTD simulations were performed in a large computational domain of size 183x183x220 mm^3^ encompassing most of the head and discretized with resolution λ/5=0.46 mm. mSOUND simulations were performed with no modeling of reflections in both the large computational domain (‘Large FOV’) and a smaller computational domain of size 79x79x160 mm^3^ corresponding to the 2f/1.3d criterion of section 2.2 for the F=80 mm transducer (‘Reduced FOV’).

**Supplementary material: Discussion**

- 1. ***Comparison of MBN with LOST***

Another finding is that scalp maps obtained with (MBN) and without skull modeling (‘Water’ method) display a high degree of similarity (Figs. 11 & S9), indicating that the main determinants of dose delivery planning are the transducer characteristics, the shape of the subject’s head/skull and the position of the target nucleus. Skull effects are smaller than those geometrical considerations, yet cause subtle changes that can significantly affect the optimal transducer position – which is why MBN outperforms ‘Water’ in all scenarios (Fig. 6). A computationally effective strategy could be to compute approximate scalp maps using the ‘Water’ method, and then refine the beam calculation with full skull modeling for the most promising transducer positions only. This would reduce the total computation time but would produce less general results, which, in view of the moderate computation time associated with MBN, may only be a marginal improvement. Another limitation of the ‘Water’ approach is that, like LOST, it does not allow comparison of the dose across subjects, whereas MBN does.

- 1. ***Computational times and requirements***

A limitation of our approach is that it computes beam solutions for a discrete set of virtual transducers. As a result, intermediate transducer positions located between scalp mesh faces must be evaluated at the closest solved transducer position. In our experience, this is not a major limitation when using ~4000 faces in the scalp mesh or more, as such a mesh density corresponds to an average distance of 6.5 mm between solved positions and the dose does not change significantly over such distance (this is shown in Figs. S1 and S2, where more than doubling the number of virtual transducers from 4,160 to 11,145, which is equivalent to reducing the distance between solved location from d=6.5 mm to d=4.0 mm, does not affect the appearance of smoothed scalp maps, nor the optimal transducer placement).

- 1. ***Smoothing of scalp maps***

In this work, we smoothed scalp maps as we found that this stabilizes the navigation experience. Indeed, smoothed scalp maps provide consistent optimal transducer positions when increasing the scalp mesh resolution, whereas raw scalp maps do not (Fig. S1 & S2). The jaggedness of raw scalp maps is due to the fact that small variations of the transducer normal can result in large displacements of the hotspot, especially when using transducers with a long focal distances. Such rapid variations of the dose across the scalp can occur in reality, but those predictions should be interpreted with caution. Instead, we recommend that the operator focuses on broad regions associated with consistently high dose deposition, for several reasons. First, we estimate scalp normals using CT or MRI volumes (whichever is used to create the head mask), which does not perfectly match experimental conditions since scalp flesh is soft and can deform when strapping the transducer to the subject’ head. Second, optical tracking has an overall accuracy of 2 mm and 1°, errors that are dominated by registration inaccuracies of the subject’ scalp to the MRI anatomical data. The Localite system uses ~500 fiducial registration points for the registration process and the overall tracking errors reported here are based on our experience and match published reports^108,109^. Smoothing scalp maps is a simple way to stabilize the navigation process in the face of those errors. A full study of tracking error propagation in MBN would be helpful to put our simulated predictions in context (confidence interval), but is beyond the scope of the present study.

- 1. ***Validation of MBN predictions***

As an illustration, Leung et al. used HAS to compute the amplitudes and phases of the InsighTech 4000 ExAblate phased-array for acoustic focusing in 9 patients with essential tremor, and found that CT-based acoustic modeling yields accurate hotspot position estimates (<1.5 mm error) but inaccurate temperature elevation predictions^32^. Ultimately, it will be necessary to demonstrate that MBN yields greater target engagement than LOST. Such work is beyond the scope of the present study as this represents a considerable effort due to the difficulty of measuring tFUS target engagement in humans. Thus, validation of MBN will likely occur hand-in-hand with studies aiming to characterize the neuromodulatory effects of tFUS using a variety of neuroimaging tools such as functional MRI, EEG and others, which is why we focus on dissemination and deployment of our tool. Another way to validate our predictions would be to measure acoustic beams *in vivo* using MRI thermometry or acoustic radiation force imaging (ARFI). Doing so is difficult however, because conventional MR thermometry and ARFI sequences can only detect large thermal and mechanical effects, and it is not clear that current sequences can detect low intensity tFUS effects.

**Supplementary material: Figures**


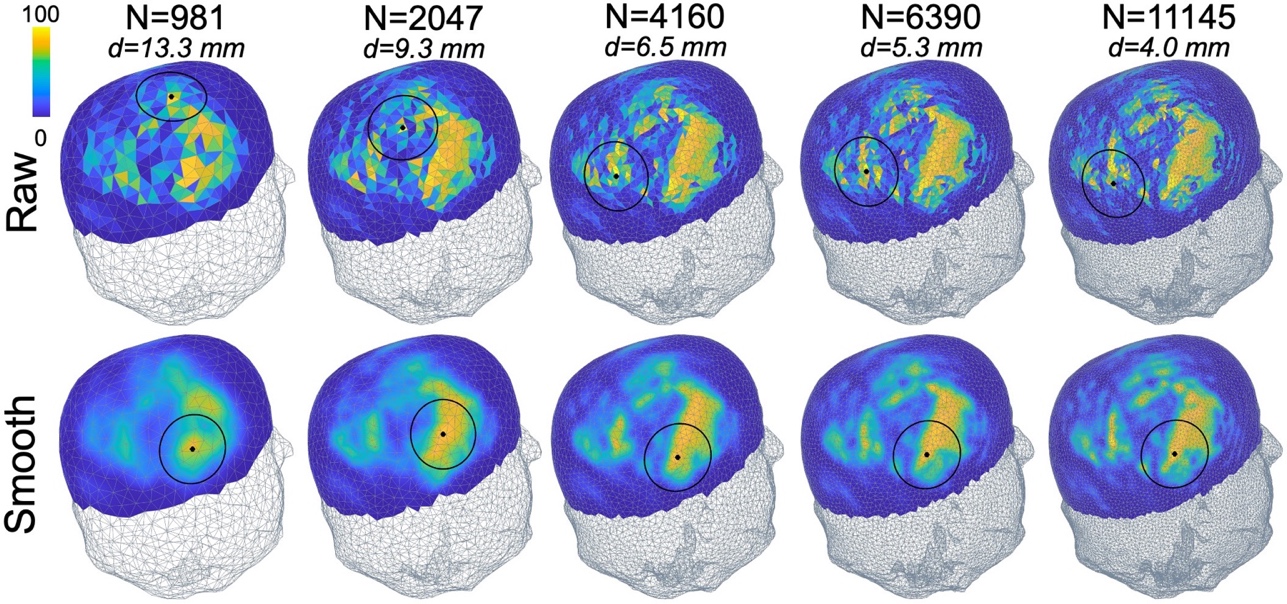


Supplementary Figure S1. Effect of mesh density and smoothing on acoustic intensity dose scalp maps of the right thalamus for subject #1 (MBN calculation, F=80 mm). N is the number of faces of the scalp mesh (=number of virtual transducers), d is the average distance between virtual transducers. Optimal transducer placements corresponding to the maximum of each scalp map’ are shown in black.


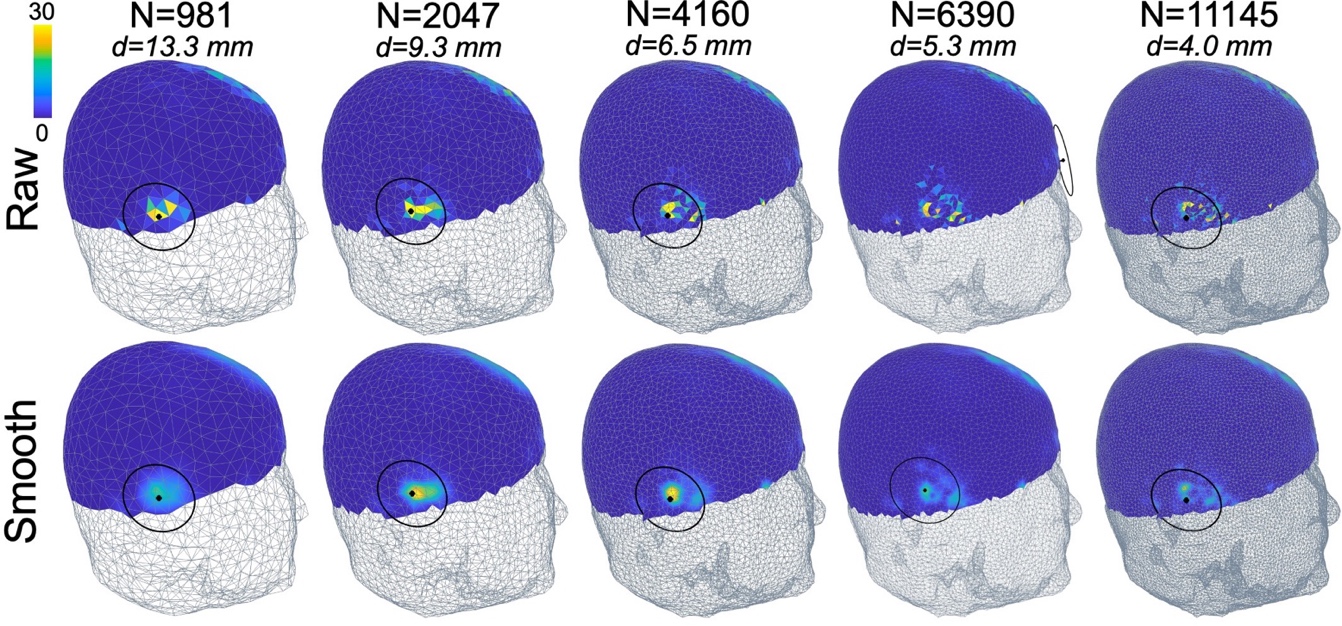


Supplementary Figure S2. Effect of mesh density and smoothing on acoustic intensity dose scalp maps of the right amygdala for subject #1 (MBN calculation, F=80 mm). N is the number of faces of the scalp mesh (=number of virtual transducers), d is the average distance between virtual transducers. Optimal transducer placements corresponding to the maximum of each scalp map’ are shown in black.


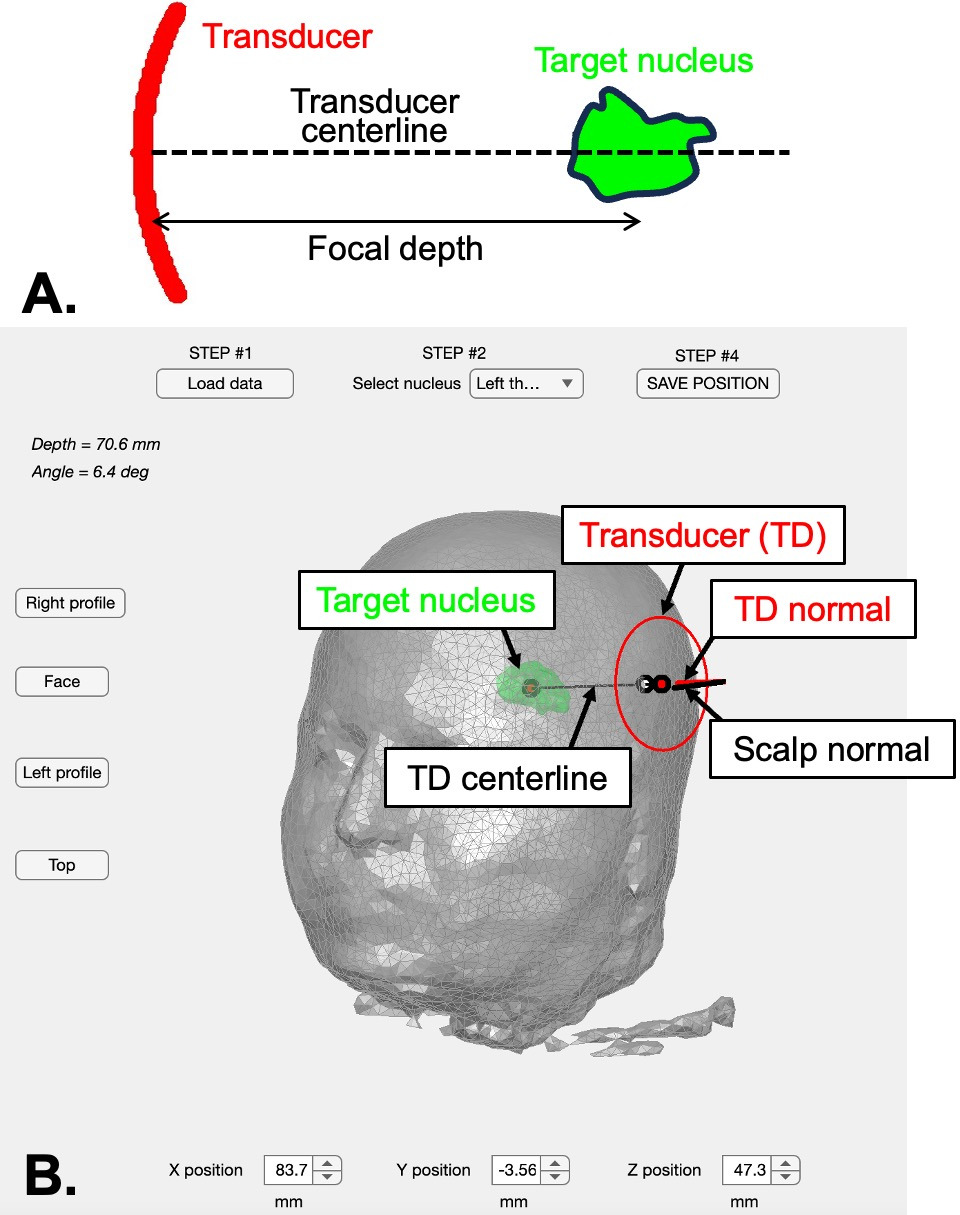


Supplementary Figure S3. **A:** Line-of-sight targeting (LOST) consists in aligning the centerline of the transducer (black) on the target nucleus (green) at the water focal depth. This alignment method assumes that the acoustic wave front propagates in a straight line from the transducer to the target, and thus ignores scattering by the skull. **B:** LOST GUI. This GUI facilitates alignment of the transducer (red) and the target nucleus (green) in our simulations. The transducer normal (red arrow), the scalp normal (black arrow) and the angle between the two are visualized to ensure that the transducer is roughly perpendicular to the scalp at the beam entry point (incident angle ≤10º).


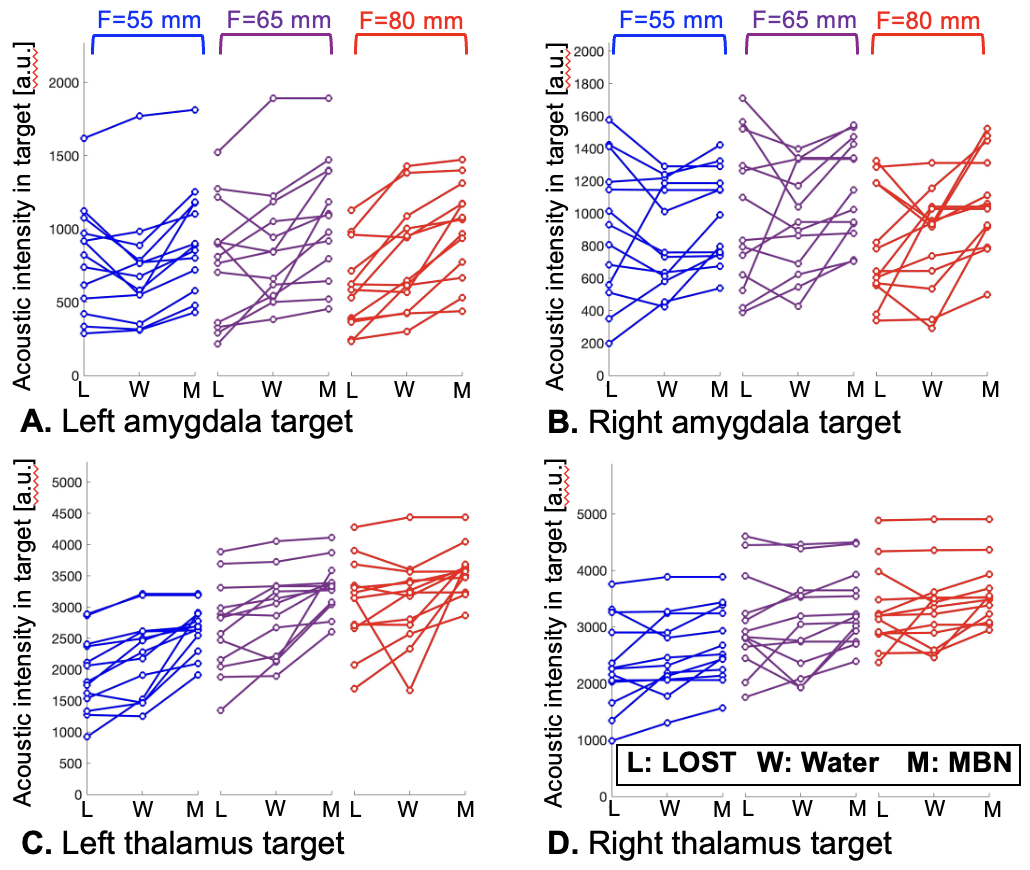


Supplementary Figure S4. Line plots showing changes of the acoustic dose in the left amygdala (A), right amygdala (B), left thalamus (C) and right thalamus (D) in individual subjects using line-of-sight targeting (LOST), water simulation (‘Water’) and model-based navigation (MBN) and transducers with focal distances F=55 mm, F=65 mm and F=80 mm.


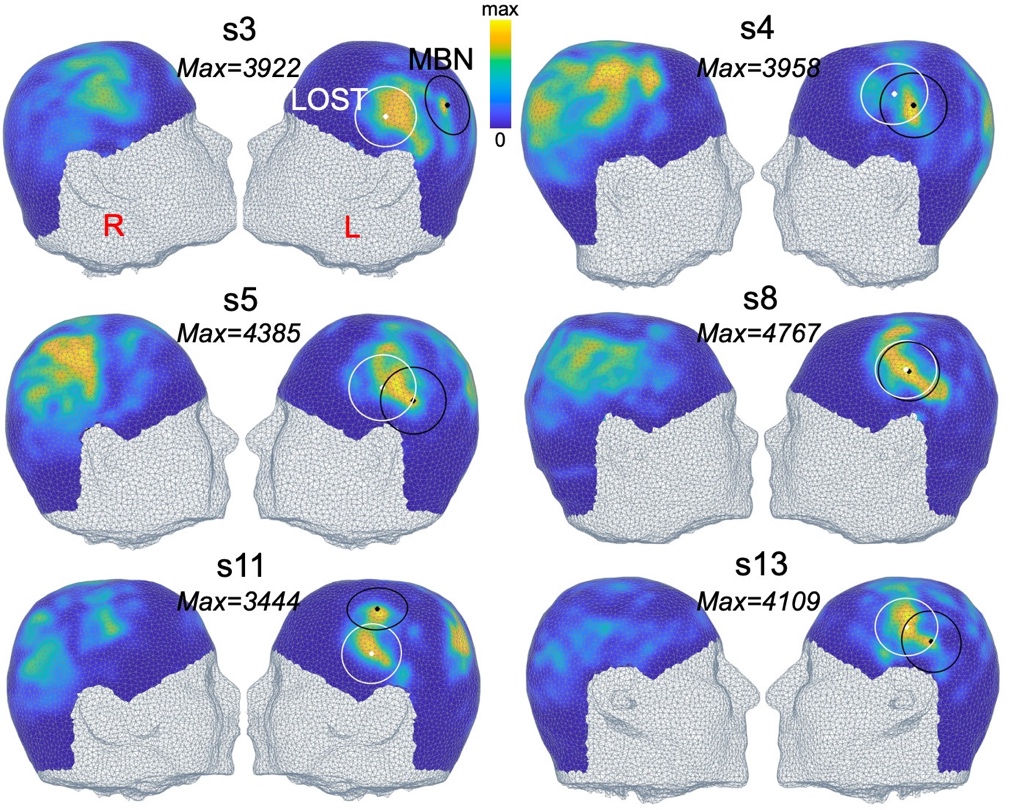


Supplementary Figure S5. Model-based navigation (MBN) scalp maps targeting the left thalamus with the F=80 mm transducer. The value next to each map indicates the peak acoustic dose deposition at the optimal MBN transducer position. Colormaps are scaled to that value. Optimal MBN transducer locations are shown in black, LOST locations in white.


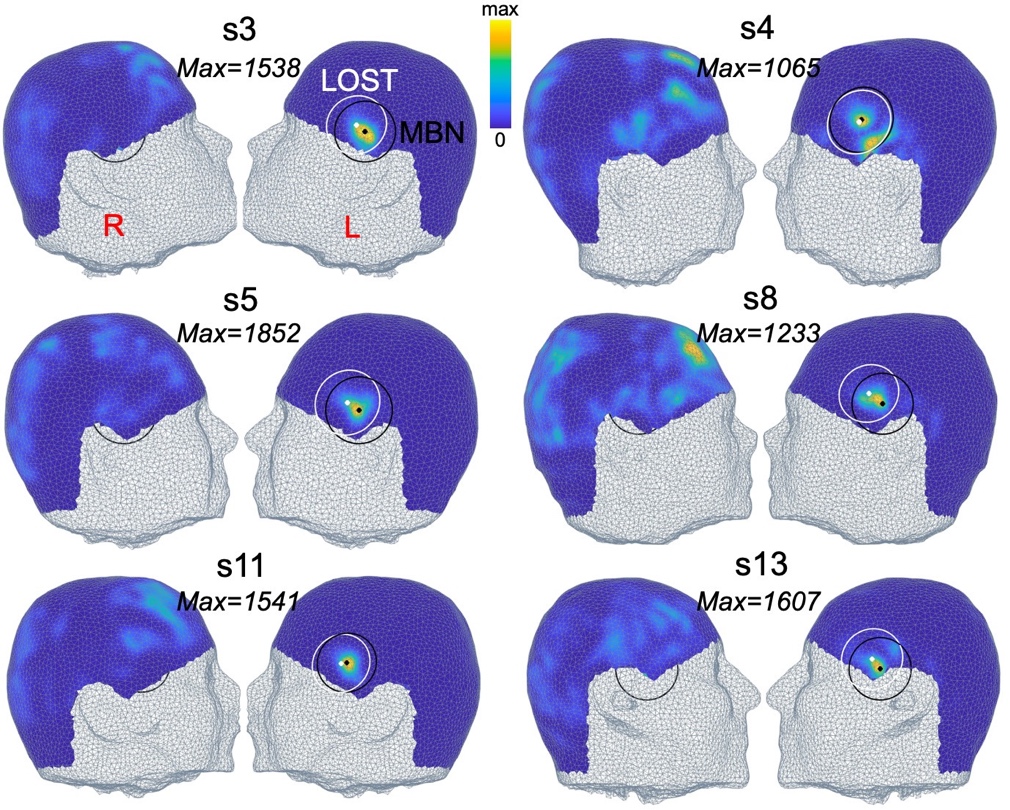


Supplementary Figure S6. Model-based navigation (MBN) scalp maps targeting the left amygdala with the F=80 mm transducer. The value next to each map indicates the peak acoustic dose deposition at the optimal MBN transducer position. Colormaps are scaled to that value. Optimal MBN transducer locations are shown in black, LOST locations in white.


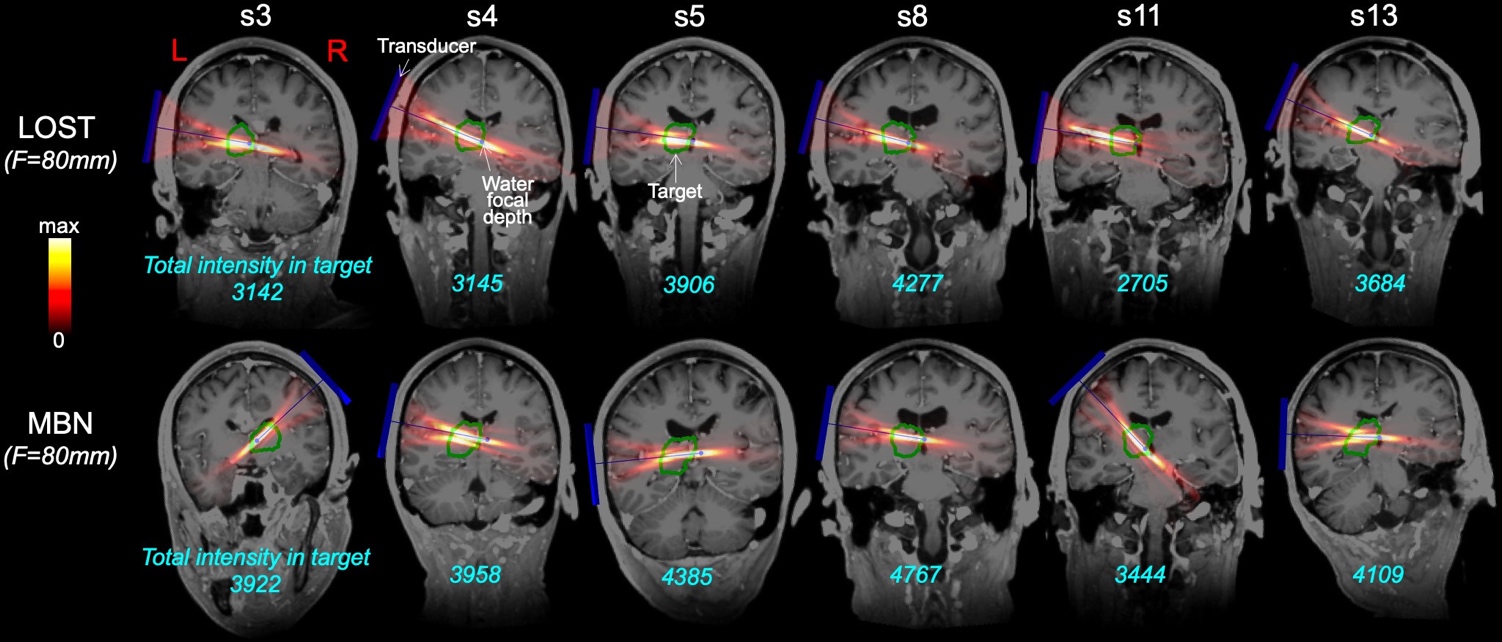


Supplementary Figure S7. TFUS beams targeting the left thalamus, overlaid on T1-images for 6 of the 13 subjects in this study. The first and second rows show beam solutions associated with line-of-sight targeting (LOST) and model-based navigation (MBN), respectively. The transducer is shown in dark blue (the transducer centerline ends in a small dot indicating the water focal depth, which is F=80 mm both for LOST and MBN since this is the optimal configuration for this target as shown in Figs. 4 & 5). The outline of the target nuclei is in green. The cyan numbers indicate the dose deposited in the target region in arbitrary units.


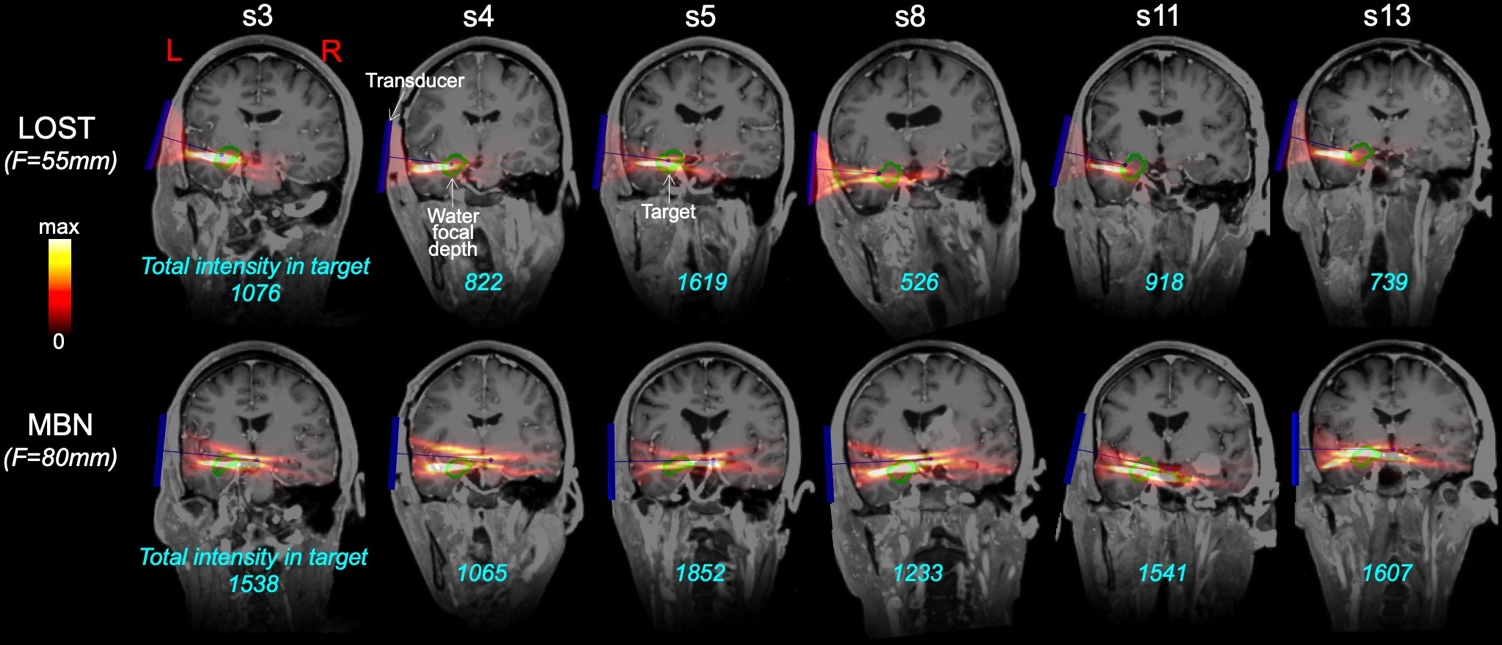


Supplementary Figure S8. TFUS beams targeting the left amygdala, overlaid on T1-images for 6 of the 13 subjects in this study. The first and second rows show beam solutions associated with line-of-sight targeting (LOST) and model-based navigation (MBN), respectively. The transducer is shown in dark blue (the transducer centerline ends in a small dot indicating the water focal depth, which is F=55 mm for LOST and F=80 mm for MBN since these are the optimal configurations for this target as shown in Figs. 4 & 5). The outline of the target nuclei is in green. The cyan numbers indicate the dose deposited in the target region in arbitrary units.


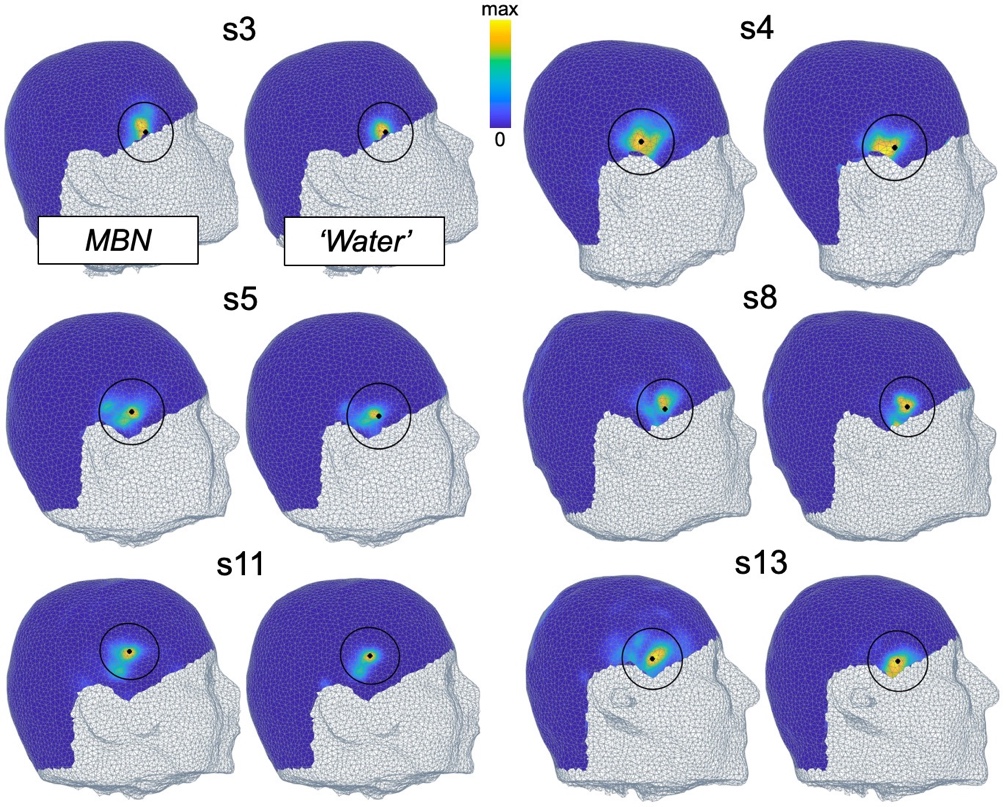


Supplementary Figure S9. Scalp maps of the dose in the right amygdala of 6 of the 13 subjects, obtained with modeling of the skull (model-based navigation, MBN) and without modeling of the skull (water simulation, ‘Water’). In the ‘Water’ approach, the ideal beam profile of the transducer in water is overlapped onto the geometry of the head and brain of the subject for all virtual transducers. Therefore, ‘Water’ can be viewed as a generalization of LOST. The transducer modeled has focal distance F=80 mm. Optimal transducer locations corresponding to the maximum of those maps are in black.


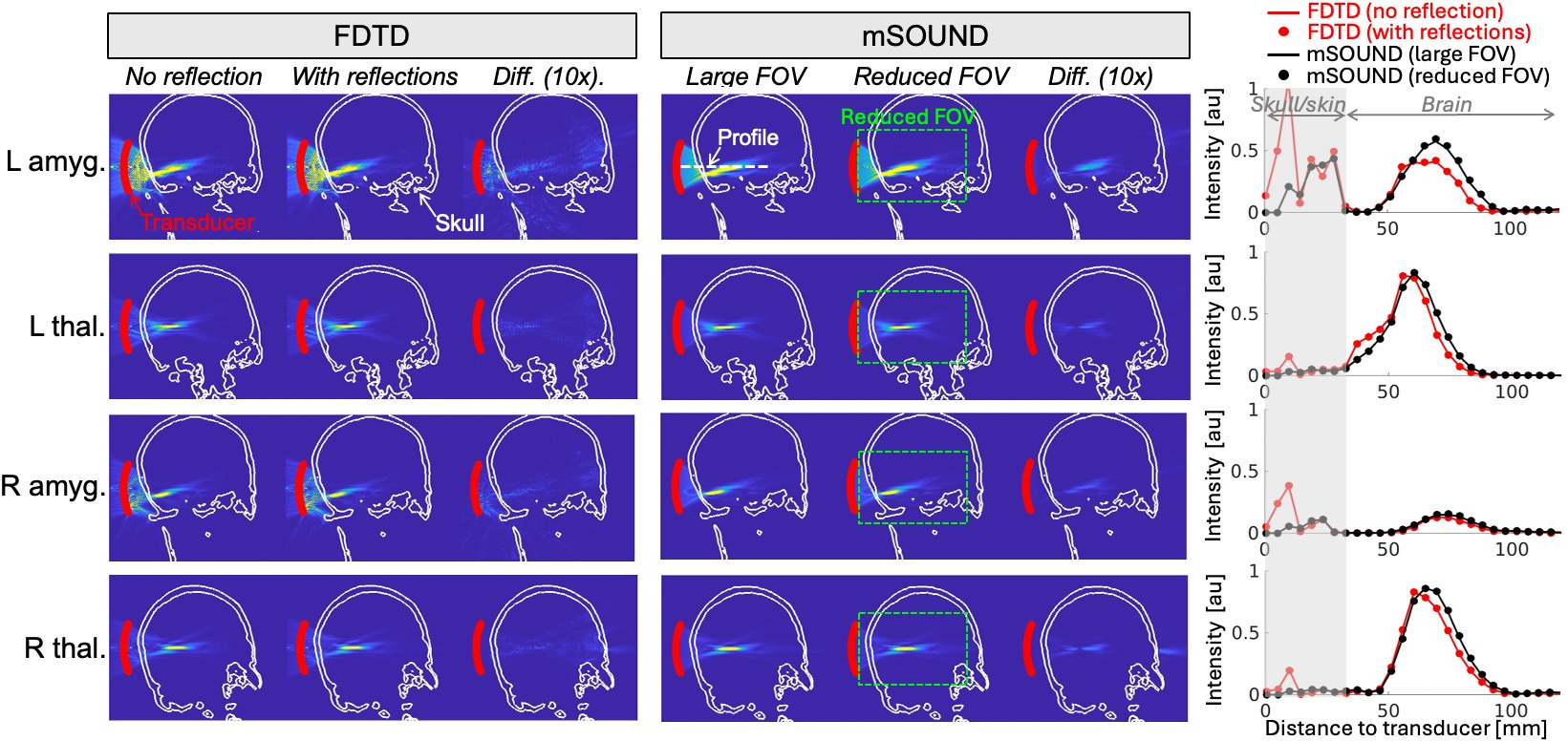


Supplementary Figure S10. Comparison between FDTD and mSOUND. **FDTD:** Simulations were performed with the LOST transducer positions targeting the L/R amygdala and thalamus in subject #1 (F=80mm transducer, 650 kHz). ‘No reflections’: The FDTD simulation duration was adjusted so as not to model any internal skull reflections. In this case, the simulation stops when the wave reaches the far wall of the skull, which took T=107 μs for this subject. ‘With reflections’: The FDTD simulation duration was adjusted so as to model up to three internal skull reflections. FDTD propagation duration was adjusted to allow for two round trips of the wavefront in the skull cavity, which took T=428 μs for this subject. There is little difference between the ‘No reflection’ and ‘With reflection’ simulations, indicating that internal skull reflections do not significantly affect estimates of the hotspot shape and position and 650 kHz. **mSOUND:** mSOUND simulations were performed without skull reflections (single forward pass through the medium, no modeling of reflected waves) in a large computational domain equal to that used in the FDTD simulation (‘Large FOV’) as well as a smaller computational domain shown in green (‘Reduced FOV’). There is little difference between the ‘Large FOV’ and the ‘Reduced FOV’ simulations, indicating that increasing the size of the mSOUND computation domain beyond that strictly containing the beam hotspot is not necessary. **Profiles:** The profiles on the right show direct comparisons between FDTD ‘No reflection’, FDTD ‘With reflection’, mSOUND ‘Large FOV’ and mSOUND ‘Reduced FOV’. There is a reasonable agreement between those simulations in the brain region, showing that fast mSOUND simulations with no reflections and a small computational domain are acceptable for estimation of the optimal transducer position at 650 kHz.
